# Green synthesis of 1,5-dideoxy-1,5-imino-ribitol and 1,5-dideoxy-1,5-imino-dl-arabinitol from natural d-sugars over Au/Al_2_O_3_ and SO_4_^2−^/Al_2_O_3_ catalysts

**DOI:** 10.1038/s41598-021-96231-9

**Published:** 2021-08-19

**Authors:** Hongjian Gao, Ao Fan

**Affiliations:** Western Digital Company, 5601 Great Oaks Parkway, San Jose, CA 95119-1003 USA

**Keywords:** Catalysis, Carbohydrate chemistry

## Abstract

A green synthetic route for the synthesis of some potential enzyme active hydroxypiperidine iminosugars including 1,5-dideoxy-1,5-imino-ribitol and 1,5-dideoxy-1,5-imino-dl-arabinitol, starting from commercially available d-ribose and d-lyxose was tested out. Heterogeneous catalysts including Au/Al_2_O_3_, SO_4_^2−^/Al_2_O_3_ as well as environmentally friendly reagents were employed into several critical reaction of the route. The synthetic route resulted in good overall yields of 1,5-dideoxy-1,5-imino-ribitol of 54%, 1,5-dideoxy-1,5-imino-d-arabinitol of 48% and 1,5-dideoxy-1,5-imino-l-arabinitol of 46%. The Au/Al_2_O_3_ catalyst can be easily recovered from the reaction mixture and reused with no loss of activity.

## Introduction

Iminosugars are analogues of carbohydrates, chemically named as polyhydroxylated secondary and tertiary amines and found to be widespread in plants and microorganisms. Thanks to their structural similarity to sugar molecules and excellent metabolic stability, iminosugars are endowed with a high pharmacological potential for a wide range of diseases such as viral infections, tumor metastasis, AIDS, diabetes and lysosomal storage disorders^1–11^.

Iminosugars are generally classified into five structural classes: pyrrolidines, piperidines, indolizidines, pyrrolizidines and nortropanes^12^. Hydroxypiperidines are structurally six-membered iminosugars. Some of the hydroxypiperidines such as 1,5-dideoxy-1,5-iminohexitol derivatives have now been commercialized as drugs to treat type II diabetes mellitus, type I Gaucher disease, Niemann-Pick disease type C (NP-C) and Fabry disease^13–18^. Other Hydroxypiperidines like 1,5-Dideoxy-1,5-imino-ribitol and 1,5-Dideoxy-1,5-imino-arabinitol derivatives have also attracted considerable attention as enzyme inhibitors that mimic glycoside and nucleoside substrates. For example, 1,5-dideoxy-1,5-imino-ribitol derivatives was found to be a potent inhibitor of bovine β-galactosidases and almond β-glucosidase^19^, while 1,5-Dideoxy-1,5-imino-arabinitol N-carboxypentyl derivatives permitted the isolation of pure α-l-fucosidase from bovine kidney homogenate^20^.

**Figure 1:**
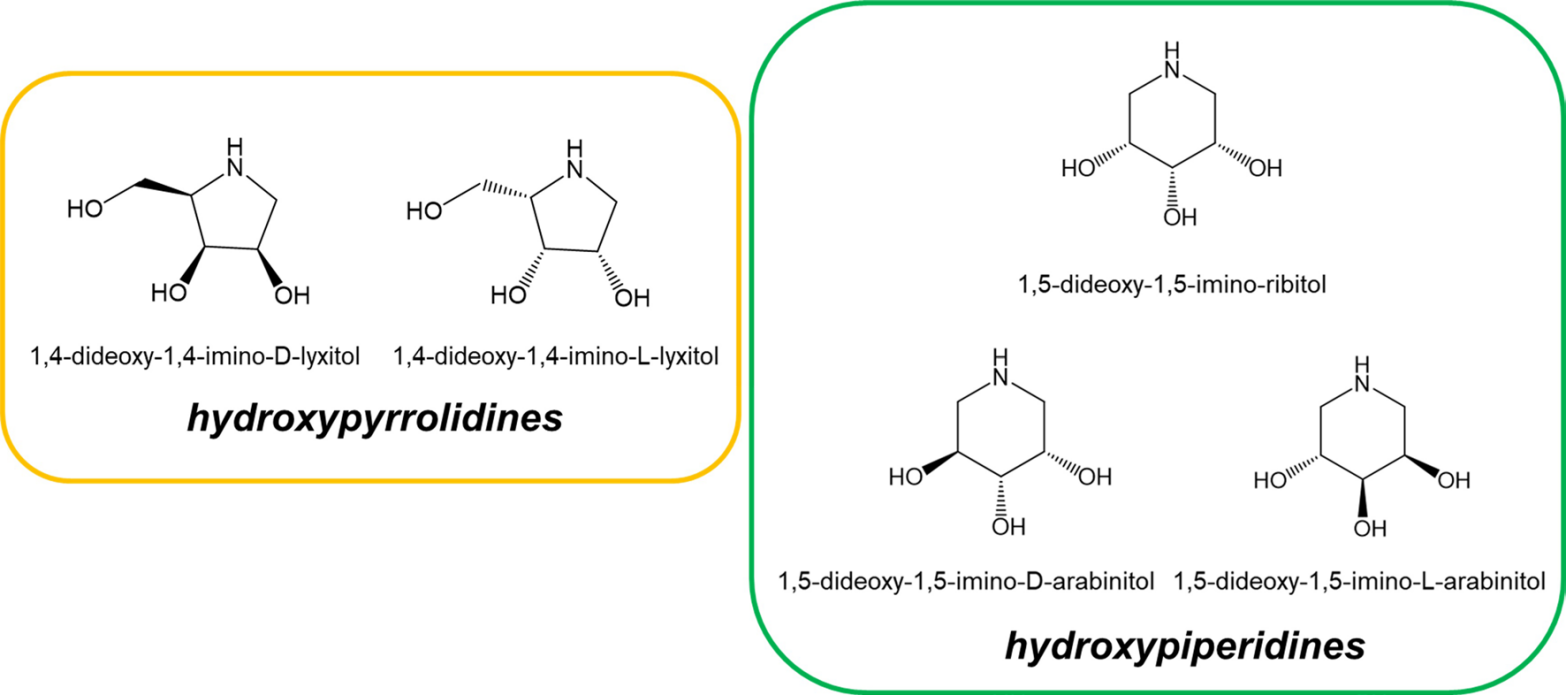
Structures of some hydroxypyrrolidine and hydroxypiperidine iminosugars.

**Scheme 1:**
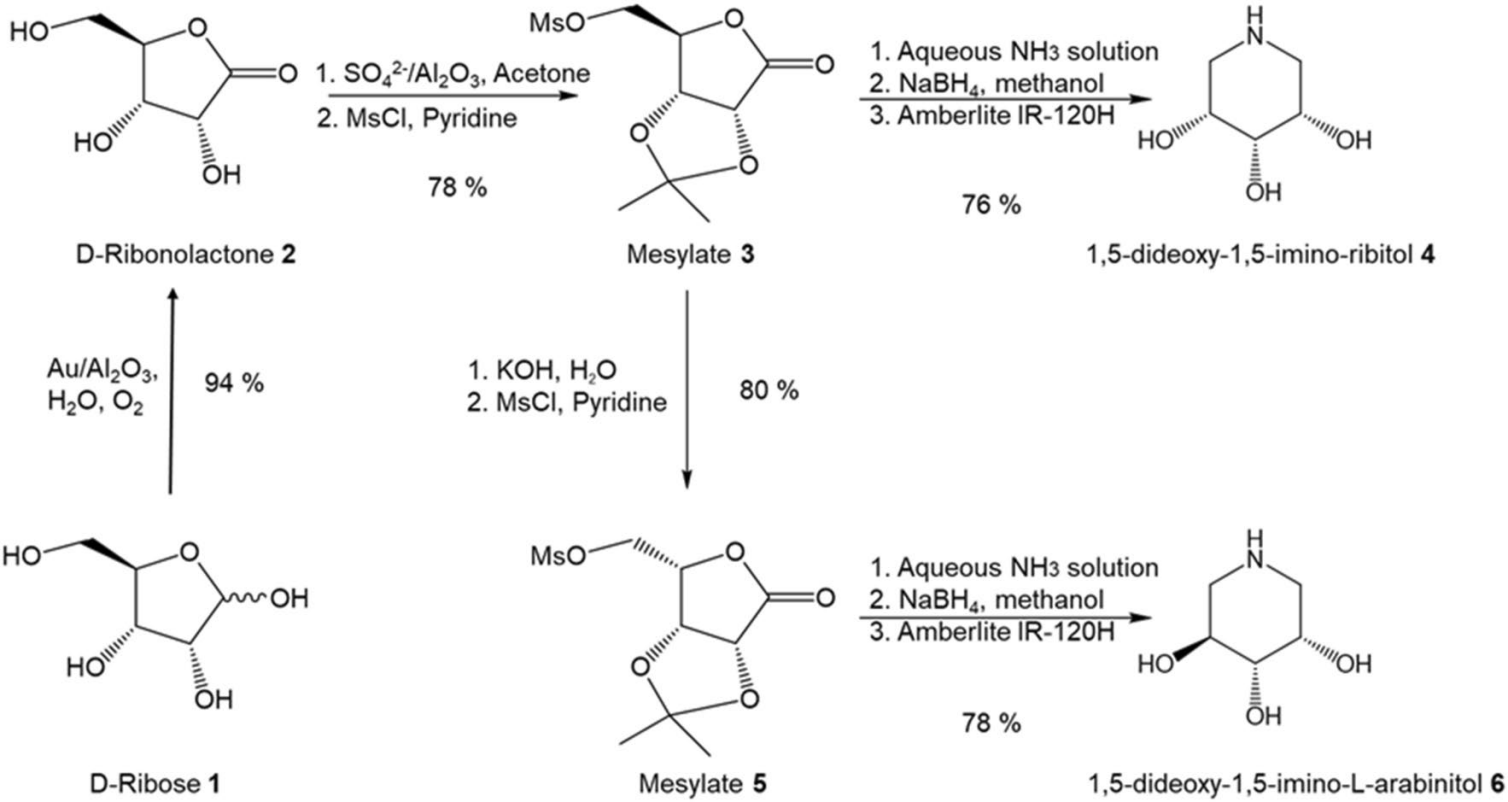
Synthesis route from d-ribose to 1,5-dideoxy-1,5-imino-d-ribitol 4 and 1,5-dideoxy-1,5-imino-l-arabinitol **6.**

There are a diversity of synthetic methodologies have been developed to access iminosugars in hydroxypyrrolidines series^21–25^, while the reports for hydroxypiperidine iminosugars synthesis, especial for hydroxypiperidine of pentitols, are limited^26^. Therefore, there is a need to develop a simple method for the preparation of 1,5-Dideoxy-1,5-imino-ribitol and 1,5-Dideoxy-1,5-imino-arabinitol (Fig. 1). In this report, we like to present an efficient and environmentally friendly route for 1,5-dideoxy-1,5-imino-ribitol and 1,5-dideoxy-1,5-imino-l-arabinitol synthesis (Scheme 1) from d-ribose. The synthetic strategy employs many principles of Green Chemistry such as avoiding toxic or noxious chemical, and recycling and reusing the reagents^27^_._

**Scheme 2:**
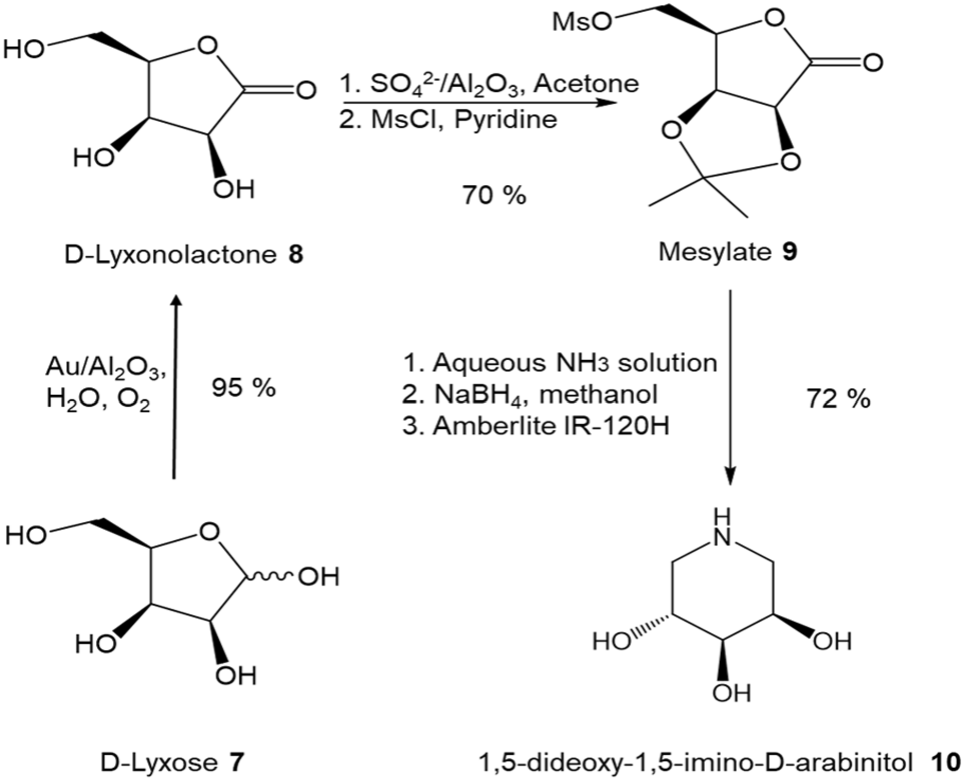
Synthesis route from d-ribose to 1,5-dideoxy-1,5-imino-d-arabinitol **10.**

The oxidation of d-ribose to d-ribonolactone is the critical and also the most difficult reaction in our proposed route. Pd–Bi/C heterogeneous catalyst^28^ with molecular oxygen has been successfully applied to directly convert d-ribose to d-ribonolactone. However, Bi–gluconate complexes formed due to the interaction between the leached Bi (from Pd-Bi/C catalyst) and glucose substrate would contaminate the gluconic acid products. As a result, further purification of products are needed, introducing large amounts of metal pollution^29^. Additionally, to prevent the catalyst poisoning due to the adsorption of the product during reaction, the reaction medium has to be maintained at pH 9.0 by continual addition of alkaline solution. Thus, the alkaline systems induce plenty of wastes (e.g., inorganic salts). Hence, the alkaline-free direct oxidation of d-ribose over heterogeneous catalysts is an ideal green process to pursue. Recently, Song Guo et al*.*^30^ reported a simple incipient wetness protocol to prepare ultra-small gold clusters on TiO_2_ through using anthranilic acid as a stabilizing agent. The resultant Au/TiO_2_ catalyst exhibits excellent catalytic activity in the alkaline-free oxidation of glucose. Because of the excellent material physical properties of γ-Al_2_O_3_ such as high thermal stability, large surface area, and superior mechanistic strength, highly mesoporous γ-Al_2_O_3_ has been widely used in the chemical industry as catalyst support. Moreover, there are abundant of cation vacancies on γ-Al_2_O_3_ surface^31^. This unique surface structure of γ-Al_2_O_3_ helps to stabilize Au clusters and prevent clusters agglomeration on its surface^32^. Thus, we decided to explore ultra-small gold clusters on γ-Al_2_O_3_ (Au/Al_2_O_3_) as catalyst for alkaline-free oxidation of d-ribose. To achieve small gold particles (2–3 nm) deposited on Al_2_O_3_ support, a simple solid grinding method was used in catalyst preparation^33^. The applicability of the Scheme 1 to the synthesis of 1,5-dideoxy-1,5-imino-d-arabinitol **10** starting from d-lyxose was also attempted (Scheme 2).

## Results and discussion

### Catalyst characterization

**Figure 2:**
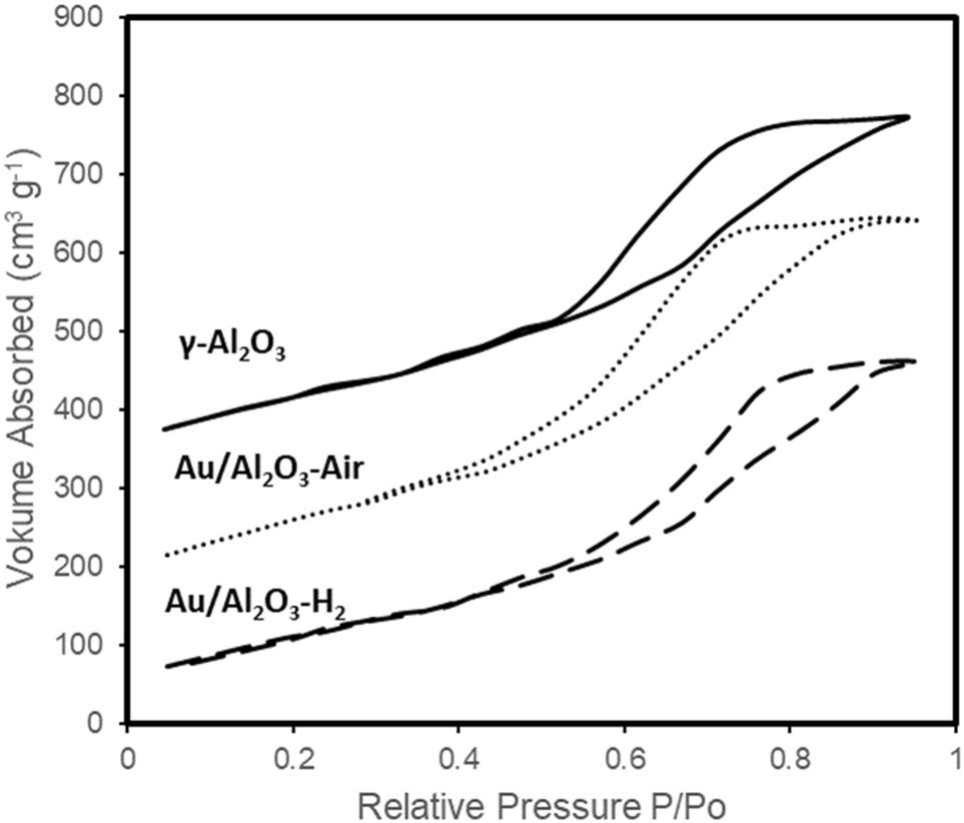
Adsorption/desorption isotherms of γ-Al_2_O_3_ and Au/Al_2_O_3_ samples.

The Au/Al_2_O_3_ catalysts were prepared through the solid grinding method^33^. The Al_2_O_3_-supported gold clusters were calcined in static air at 300 °C, and the sample is denoted Au/Al_2_O_3_-Air. Prior to use, the catalysts were reduced under H_2_ flow for 2 h at 150 °C and denoted Au/Al_2_O_3_-H_2_. The nitrogen adsorption–desorption isotherms (Fig. 2) of catalyst samples with type IV shape designated the presence of mesopores with uniform pore size distribution^34^. Table 1 presents the composition, BET surface area, pore volume, average pore diameter of γ-Al_2_O_3_ support and Au/Al_2_O_3_ catalysts. The BET surface area, pore volume and average pore diameter were found to be decrease slightly with the loading of Au content, showing that Au was deposited into the pores of γ-Al_2_O_3_ support.

**Table 1:** Textural properties of γ-Al_2_O_3_ and γ-Al_2_O_3_ supported nano Au catalysts.

Catalyst	Surf. area (m^2^ g^−1^)	Pore vol. (m^2^ g^−1^)	Ave. pore. diameter (nm)	Au content (wt %)^a^
γ-Al_2_O_3_	176	0.62	6.14	–
1 wt.% Au/Al_2_O_3_-Air	161	0.59	5.53	0.93
1 wt.% Au/Al_2_O_3_-H_2_	156	0.58	5.49	0.91

^a^Based on ICP-OES analysis.

**Figure 3:**
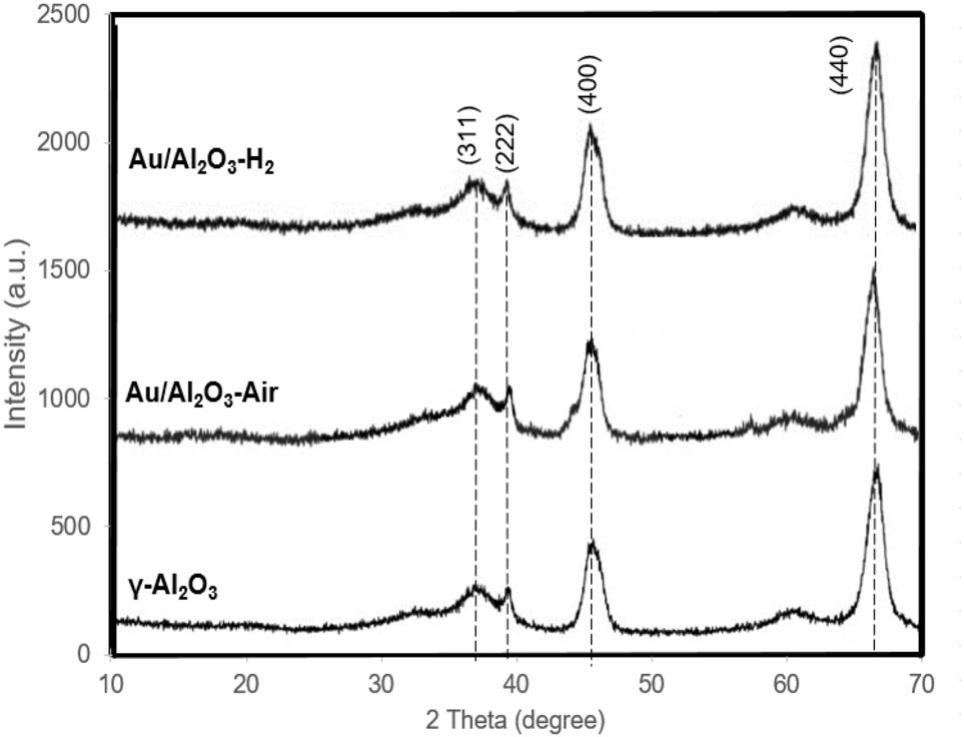
XRD spectra of γ-Al_2_O_3_ and Au/Al_2_O_3_ samples.

XRD and TEM were then used on analysing these catalysts to determine the differences in the size and distribution of gold particles on γ-Al_2_O_3_ support. As shown in Fig. 3, no reflections associated with Au nanoparticles were found on XRD patterns of the Au/Al_2_O_3_. Actually, the diffraction pattern Au/Al_2_O_3_ is well in accordance with γ-Al_2_O_3_ supports (JCPDS 29-63). The XRD results indicate that the gold particles are uniformly dispersed on the oxide surface.

**Figure 4:**
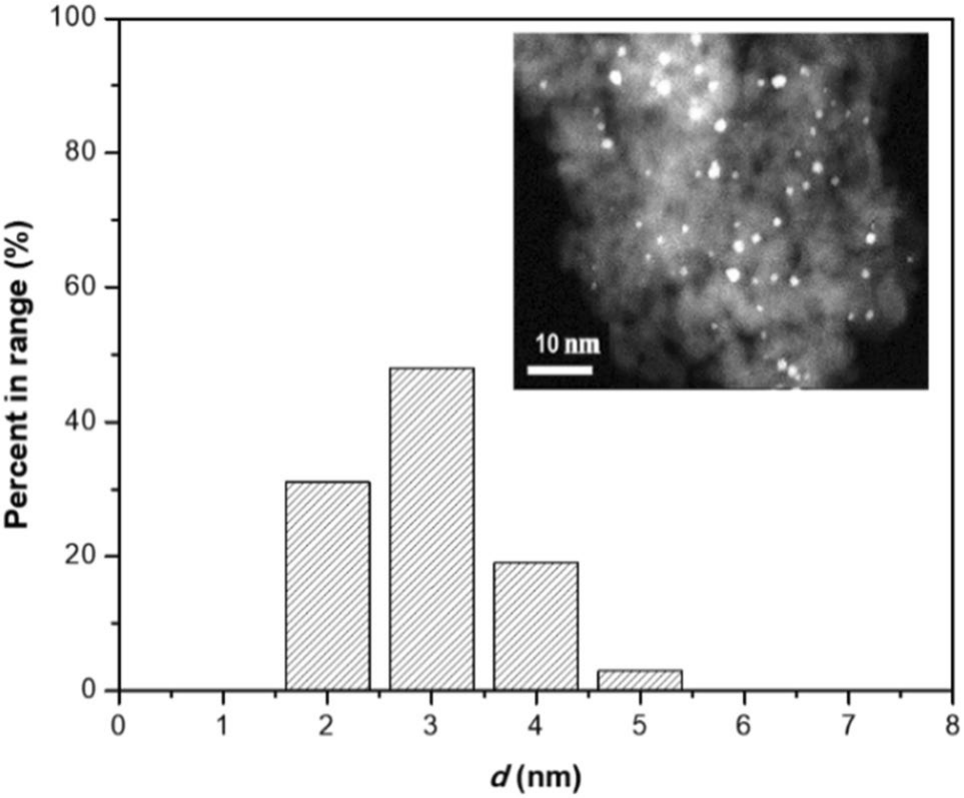
STEM image and size distribution of Au/Al_2_O_3_-H_2_ sample.

As shown in Fig. 4, the gold nano particles are uniformly distributed on Al_2_O_3_-H_2_ support. The average particle size of an Au/Al_2_O_3_-H_2_ catalyst is 2–3 nm, which was similar to particle size reported in the literature^33^. The small clusters and uniform dispersion results are in agreement with XRD analysis.

**Figure 5:**
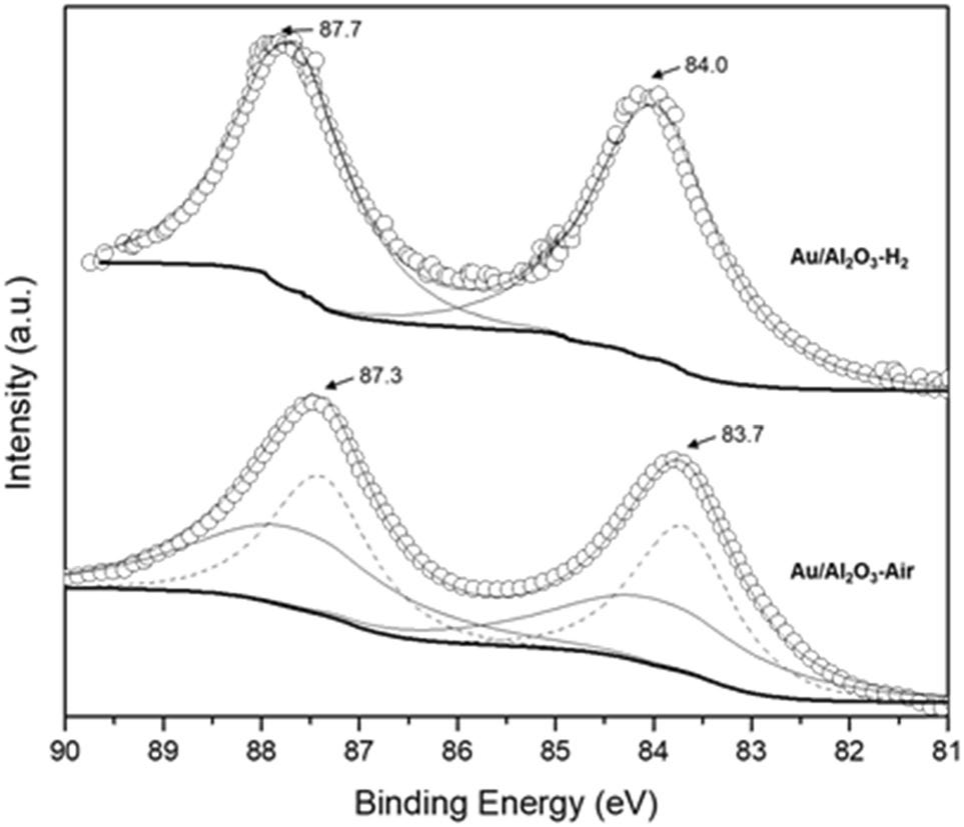
XPS spectra of γ-Al_2_O_3_ and Au/Al_2_O_3_ catalysts for Au 4f.

To understand the surface state of Au particles on γ-Al_2_O_3_ surface, the XPS Au 4f spectra were carefully examined for the catalysts before and after H_2_ reduction. Based on some XPS spectra obtained from various Au catalysts^35,36^ previously, Au 4f_5/2_ 87.7 eV and Au 4f_7/2_ 84.0 eV could be referred as neutral Au species. As shown in Fig. 5, compared with Au/Al_2_O_3_ without H_2_ reduction, H_2_ reduction reduce positive gold species and lead Au 4f peaks shift upward in binding energies (BEs) by 0.3–0.4 eV to neutral Au species. The XPS results reveal that the H_2_ reduction could help to reduce positive gold species and produce neutral Au species on the γ-Al_2_O_3_ surface.

### Synthesis of 1,5-dideoxy-1,5-imino-d-ribitol 4 and 1,5-dideoxy-1,5-imino-l-arabinitol 6 from d-ribose

#### Oxidation of d-ribose

In the first step as outlined in Scheme 1, the aerobic d-ribose oxidation was carried out in pure water using O_2_ gas (1 MPa) as an oxidant agent. We first examined the catalytic performance of conventional gold nanoparticles (Au/C of 3–5 nm Au particle size) and commercial Pd/C catalyst as well as the synthesized Pd–Bi/C catalyst^28^. It is found that the Au/C catalyst gave a 72% d-ribose conversion with a relatively low ribonolactone selectivity of 82% (Table 2, entry 2). By-products of glutarate and oxalate were formed due to overoxidation and degradation of d-ribose, respectively. The Pd/C and Pd–Bi/C catalysts showed much lower catalytic performance (45% and 36% for d-ribose conversion (Table 2, entries 3 and 4) due to catalyst poison^28^. Intriguingly, their selectivities to ribonolactone was low (≤ 20%)due to the over-oxidized targeted products. For Au/Al_2_O_3_-Air catalyst, 24% low yield with > 95% selectivity for ribonolactone was obtained. Interesting, simply pretreated the Au/Al_2_O_3_-Air under H_2_ flow for 2 h at 150 °C could significantly improve its catalytic activity, and 93% conversion with > 95% selectivity to ribonolactone (Table 2, entry 6) was achieved. Of note, Au/Al_2_O_3_ catalyst showed low activity under relatively low reaction temperature conditions (Table 2, entries 7).

**Table 2:** d-Ribose oxidation under different conditions.

Entry	Catalyst	Temp. (°C)	Conv. (%)	Sel. (%)
1	–	100	–	–
2	1 wt.% Au/C	100	72	82
3	10 wt.% Pd/C	100	45	15
4	10 wt.% Pd-Bi/C	100	36	20
5	1 wt.% Au/Al_2_O_3_-Air	100	24	> 95
6	1 wt.% Au/Al_2_O_3_-H_2_	100	94	> 95
7	1 wt.% Au/Al_2_O_3_-H_2_	70	67	> 95

Reaction conditions: 0.15 g of d-ribose in 10 mL of H_2_O, 36 mg of catalyst, 2 h, 1 MPa O_2_. The Au loading of the prepared catalysts is 1 wt.%, and ribose/Au (molar ratio) = 550/1. The conversions (conv.) of ribose and selectivity (Sel.) for ribonolactone were determined by the HPLC analysis.

Song Guo et al*.*^30^ proposed that the glucose and dioxygen molecules are adsorbed and activated on the Au^0^ sites and support, respectively. The occupancy of Au^δ+^ species at the particles surface by water solvents and oxygen species would block the nearby active Au^0^ sites for glucose and O_2_ absorption, which would result in a low catalytic performance. From the XPS results (Fig. 5), pretreating the Au/Al_2_O_3_-Air catalyst under H_2_ could almost fully reduce surface Au^δ+^ species to Au^0^ species, which helps to reduce/remove the blocking effect from Au^δ+^ species and improve the catalytic activity.

**Figure 6:**
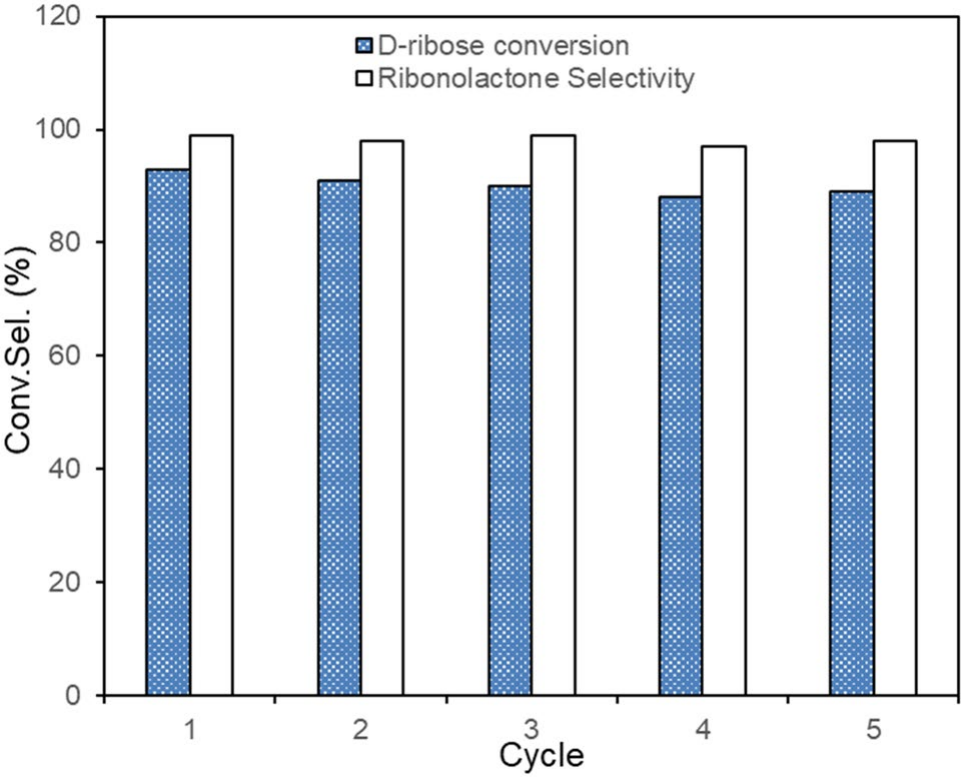
Conversion and selectivity of d-ribose oxidation with recycled Au/Al_2_O_3_ catalyst.

The recyclability of Au/Al_2_O_3_ was evaluated under the same reaction conditions using recycled catalysts. Five cycles were carried out for d-ribose oxidation at 100 °C for 2 h under 1 MPa O_2_ using a recycled Au/Al_2_O_3_ catalyst and fresh reactants. After each reaction, the catalyst was simply recovered by filtration and washed alternately with water and ethanol followed by drying before being used again. The used catalyst was tested in 5 batch reactions without loss of activity and selectivity (Fig. 6). Hence, the results showed that Au/Al_2_O_3_ is a reusable and selective catalyst for d-ribose oxidation.

#### Acetalization of d-ribonolactone to 2,3-*O*-isopropylidene-d-ribonolactone

It was found that anhydrous CuSO_4_ served as critical Lewis catalyst^28^ for the conversion of ribonolactone to 2,3-*O*-isopropylidene-d-ribonolactone. Although relatively high yield (72%) was achieved, the CuSO_4_ is hard to be fully recovered due to its excellent solubilty in water. Marek Marczewski^37^ reported a SO_4_^2−^/Al_2_O_3_ system, where the acid strength of SO_4_^2−^/Al_2_O_3_ catalyst could be tuned based on the concentration of H_2_SO_4_ solution through incipient wetness method. Therefore, we next optimized the condition of SO_4_^2−^/Al_2_O_3_ catalysts for the acetalization of ribonolactone with acetone to produce 2,3-*O*-isopropylidene-d-ribonolactone.

As shown in Table 3, no 2,3-acetonide **3a** was formed when there was no catalyst in the reaction system. The addition of anhydrous CuSO_4_ improved the yield to 68% when the reaction mixture was refluxed at 60 °C for 2 h (Table 3, entry 2). Interestingly, the surface sulfates of γ-Al_2_O_3_ created through H_2_SO_4_ solution treatment could increase 2,3-acetonide **3a** yield significantly: The yield for commercial γ-Al_2_O_3_ is only 37%, while the reactions using 1 wt.% SO_4_^2−^/Al_2_O_3_ and 3 wt.% SO_4_^2−^/Al_2_O_3_ produced 71% and 85% 2,3-acetonide **3a**, respectively. However, the use of 9 wt.% SO_4_^2−^/Al_2_O_3_ resulted in charring and only 11% yield of **3a** was obtained (Table 3, entry 7). In addition, extending the refluxing time to 4 h with 3 wt.% SO_4_^2−^/Al_2_O_3_ catalyst led to the formation of some unknown products (Table 3, entry 6).

**Table 3:** Acetalization of ribonolactone with acetone.
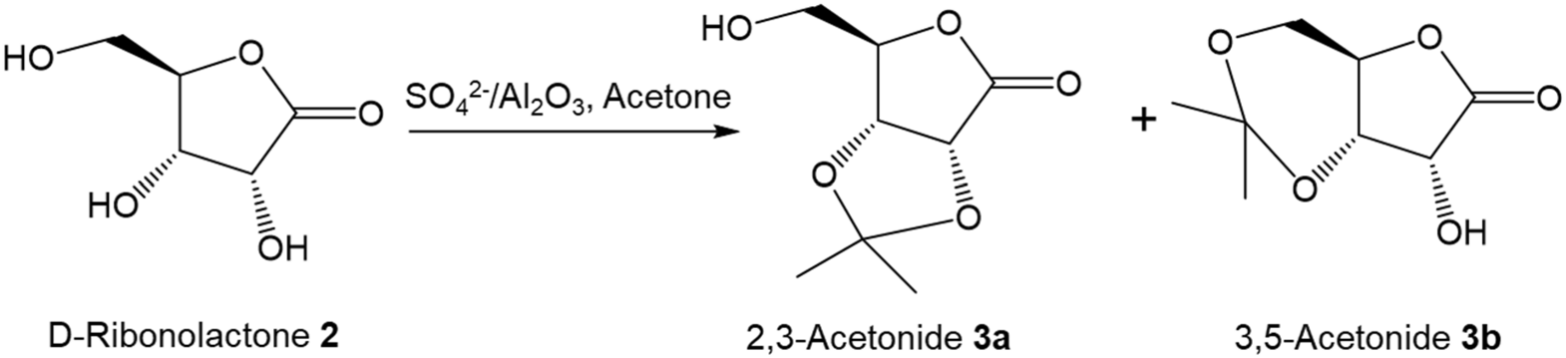

Entry	Catalyst	Super acid sites (mmol/g)^a)^	Acetone (cm^3^)	Condition	Yield **3** (%)
1	–	–	40	Reflux at 60 °C for 6 h	0
2	CuSO_4_	–	40	Reflux at 60 °C for 2 h	68
3	γ-Al_2_O_3_	0.082	40	Reflux at 60 °C for 2 h	37
4	1 wt.% SO_4_^2−^/Al_2_O_3_	0.276	40	Reflux at 60 °C for 2 h	71
5	3 wt.% SO_4_^2−^/Al_2_O_3_	0.623	40	Reflux at 60 °C for 2 h	86
6	3 wt.% SO_4_^2−^/Al_2_O_3_		40	Reflux at 60 °C for 4 h	95^b)^
7	9 wt. % SO_4_^2−^/Al_2_O_3_	1.114	40	Reflux at 60 °C for 2 h	11
8	*Recycled*^c)^ 3 wt.% SO_4_^2−^/Al_2_O_3_	0.448	40	Reflux at 60 °C for 2 h	73
9	*Re-generated*^d)^ 3 wt.% SO_4_^2−^/Al_2_O_3_	0.647	40	Reflux at 60 °C for 2 h	85

Reaction conditions: 1.5 g of crude d-ribonolactone (10 mmol) in 40 mL of acetone, 3 mmol of catalyst. ^a^Were determined by NH_3_-TPD analysis. ^b^Unknown products probably be 3,4-acetonide^38^. ^c^See Recycle procedure in SI. ^d^See Re-generation procedure in SI.

**Figure 7:**
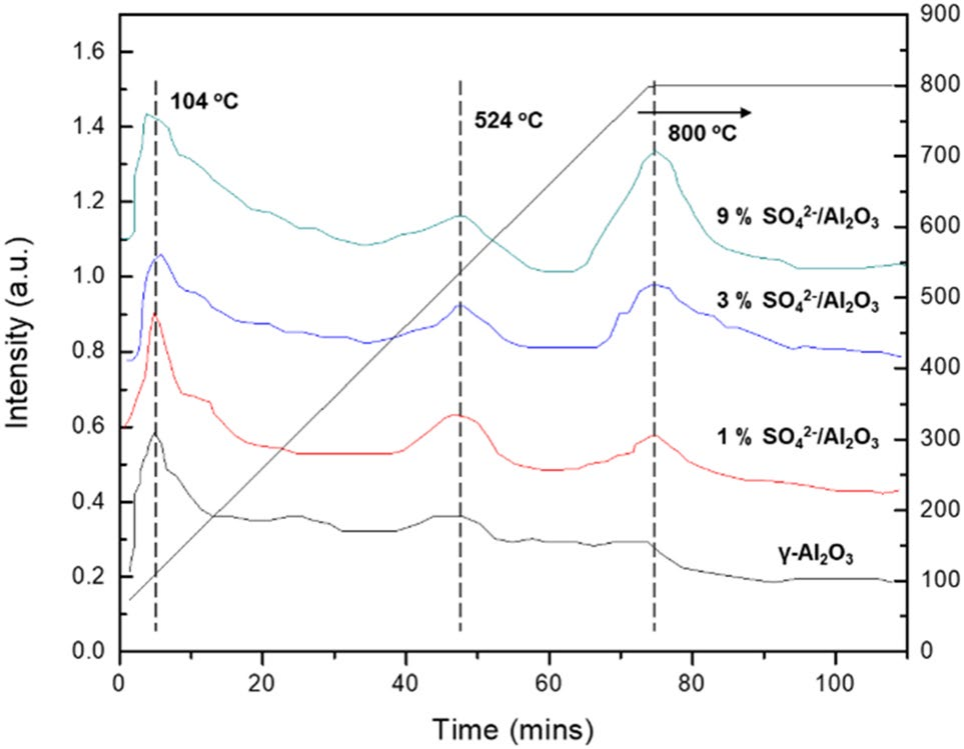
NH_3_-TPD curves of Al_2_O_3_ catalysts.

**Figure 8:**
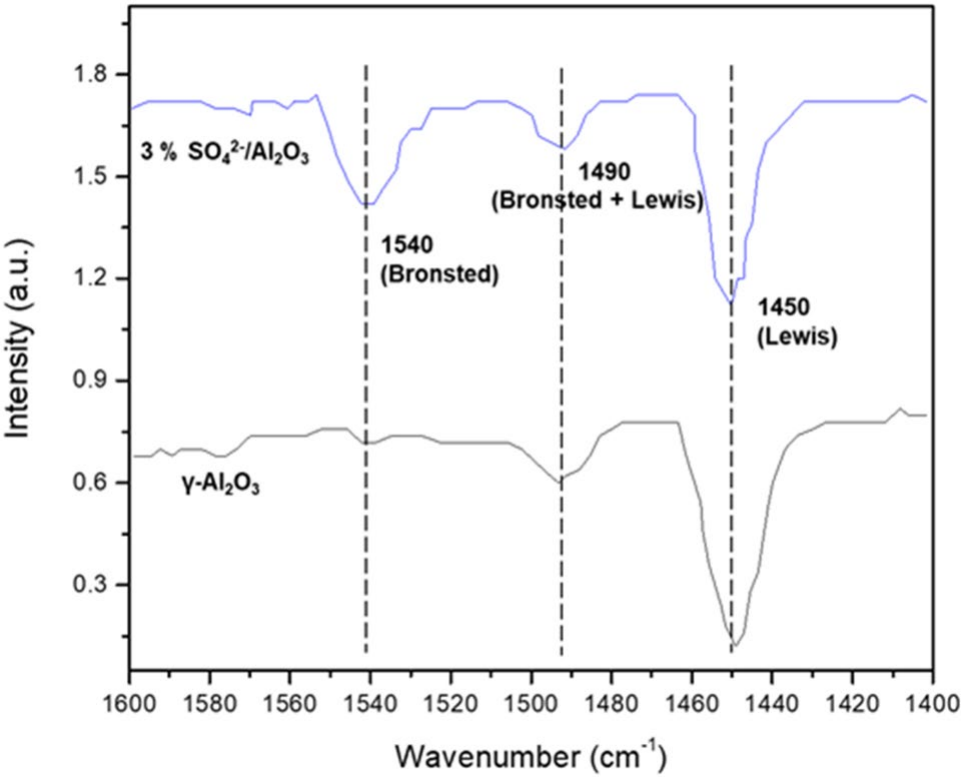
Pyridine-IR spectra of Al_2_O_3_ catalyst.

To further understand the catalytic performance of the Al_2_O_3_ samples, catalysts were examined with NH_3_-TPD and Pyridine-IR. As shown in Fig. 7, all the catalysts show two NH_3_ desorption peaks at around 104 and 524 °C, which are attributed to the weak and strong acid sites^39–41^, respectively. In addition, the third peak at around 800 °C was observed on SO_4_^2−^/Al_2_O_3_ samples. The NH_3_ desorption peak at 800 °C could be ascribed to the super strong acid sites, demonstrating the presence of super-acidic centers on SO_4_^2−^/Al_2_O_3_ catalysts. The peak at 800 °C was increased with the introduction of more SO_4_^2−^ species. Figure 8 shows the Pyridine-IR of the catalysts. Note that both Brønsted and Lewis acid are presence on SO_4_^2−^/Al_2_O_3_ catalyst, whereas the γ-Al_2_O_3_ has a very little peak at 1540 cm^−1^, indicating that there is only a small amount of Brønsted acid sites on γ-Al_2_O_3_ surface. Thus, the good catalytic activity of the 3 wt.% SO_4_^2−^/Al_2_O_3_ should be from the synergetic effect of Brønsted and Lewis acid sites as well as its appropriate amount of surface super-acid sites.

It was found that the catalytic acivities of recycled SO_4_^2−^/Al_2_O_3_ catalysts dropped significantly (Table 3, entry 9) even after re-calcination at 500 °C for 24 h under dry air. The total super acid sites of recycled 3 wt.% SO_4_^2−^/Al_2_O_3_ sample was reduced to 0.448 mmol/g comparing to 0.623 mmol/g of fresh 3 wt.% SO_4_^2−^/Al_2_O_3_, indicating the release of substantial sulfate species from Al_2_O_3_ surface in reaction (Figure S1). To recover the performance of SO_4_^2−^/Al_2_O_3_ catalyst, re-deposition of SO_4_^2−^ on spent catalyst (upto 3 wt.%) by incipient wetness method is required (Table 3, entry 10).

Treated 2,3-acetonide **3a** with methanesulfonyl chloride (MsCl) in pyridine to afford mesylate **3** as yellow crystals (91% yield)^38,42^. Mesylate **3** then was treated with aq ammonia and worked up^44^, the 2,3-*O*-isopropylidene ribonolactam could be isolated crystalline in 84% yield. The carbonyl group on the anomeric carbon of the resulting lactam was reduced with NaBH_4_ in methanol to give colorless syrup (95% yield)^28^. Finally, the resulting syrup was hydrolyzed using acidic Amberlite IR-120H resin to afford 1,5-dideoxy-1,5-imino-d-ribitol **4** in 96% yield. On the other pathway, treated mesylate **3** with potassium hydroxide to inverse the reacting carbon center and then followed by reacting with MsCl in pyridine to afford mesylate **5** in 80% yield^43^. Following the same experiment procedures (treated with aq ammonia, reduction with NaBH_4_ and hydrolysis using acidic Amberlite IR-120H) described above to obtain 1,5-dideoxy-1,5-imino-l-arabinitol **6**. Hence, through above novel and environmental friendly synthetic route, 1,5-dideoxy-1,5-imino-d-ribitol **4** and 1,5-dideoxy-1,5-imino-l-arabinitol **6** was synthesized from d-ribose with an overall yield of 54% and 46%, respectively. The yield for **4** is better than those synthesized from d-ribonolactone (38%) and allylic alcohol (30%)^43^. The yield for **6** is also significantly higher than those from using d-ribose (5–27%)^19^. and l-lyxose (8%)^44^ as starting materials. In addition, this synthetic strategy shows more environmental benefit and utilizes more principles of green chemistry than those conventional routes^19,43,44^.

The generality of this new synthetic protocol was tested for the synthesis of 1,5-dideoxy-1,5-imino-d-arabinitol **10** from d-lyxose (Scheme 2). d-lyxonolactone could be obtained by oxygenation of the far cheaper d-galactose by the Humphlett oxygenation^45^. However, the yield is low (68%) and potassium d-lyxonate instead of d-lyxonolactone was obtained, which requries extra work to convert potassium d-lyxonate to d-lyxonolactone. Different from Humphlett oxygenation, the aerobic oxidation of d-lyxose **7** with 1 wt.% Au/Al_2_O_3_ gave higher yield of desirable lactone product and > 99% conversion was achieved after 3 h. The selectivity to d-lyonolactone was > 95%.

Table 4 shows the transformation of d-lyxonolactone **8** to 2,3-*O*-isopropylidene-d-lyxonolactone **9a** under different reaction conditions. The yield for **9a** was < 5% with the use of CuSO_4_ or γ-Al_2_O_3_ in reaction (Table 4, entries 1 and 2). When the reaction mixture were refluxed over 1 and 3 wt.% SO_4_^2−^/Al_2_O_3_, the isomer 3,5-acetonide **9b** was formed. Refluxing the reaction mixture over 9 wt.% SO_4_^2−^/Al_2_O_3_ charred the reactant and products. To achieve single isomer product acetonide **9a**, the reaction mixture was stirred for 18 h at room temperature, a 43% yield of **9a** could be obtained over 3 wt.% SO_4_^2−^/Al_2_O_3_, which is significantly improved comparing with 27% yield through using methane sulfonic acid as catalyst. Fortunately, the significant difference in solubility of d-lyxonolatone **8** and 2,3-acetonide **9a** allows them to be easily separated with liquid–liquid (ethyl acetate/water) extraction. In turn, it was possible to recover the unreacted lyxonolatone and carry out further batch reactions to improve the yield. About 80% yield was achieved after three cycles of the reaction. Compound **9a** was next reacted with MsCl in pyridine solution followed by reducing the carbonyl group on the anomeric carbon of the resulting lactam with NaBH_4_ in methanol, then hydrolyzing over acidic Amberlite IR-120H resin to produce 1,5-dideoxy-1,5-imino-d-arabinitol **10**^46^. The overall yield of 1,5-dideoxy-1,5-imino-d-arabinitol **10** was 48%. The yield to d-arabinitol **10** can be compared with previously reported yields of 40% from d-arabinose^20^.

**Table 4:** Acetalization of lyxonolactone with acetone.
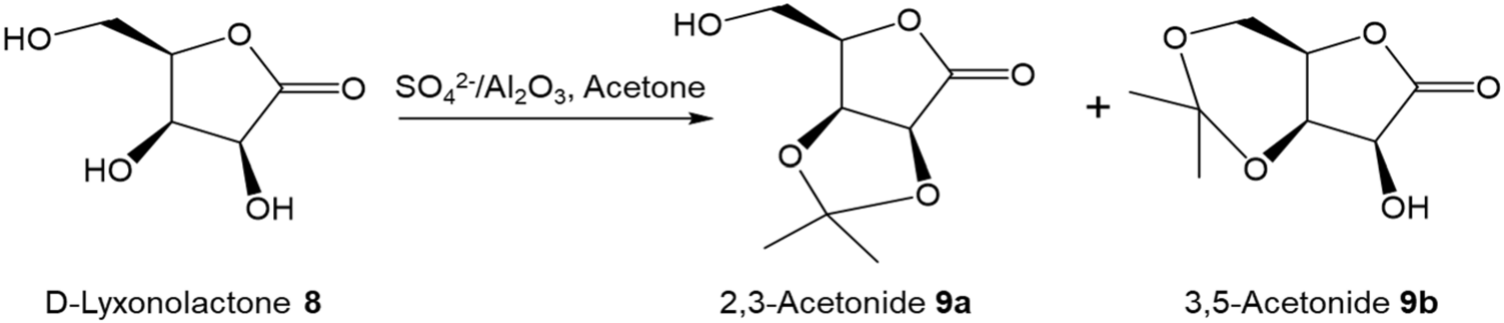

Entry	Reagents	Acetone (cm^3^)	Condition	Yield **9** (%)
1	CuSO_4_	40	Reflux for 4 h	< 5
2	γ-Al_2_O_3_	40	Reflux for 4 h	0
3	1 wt.% SO_4_^2−^/Al_2_O_3_	40	Reflux for 4 h	33^a^
4	3 wt.% SO_4_^2−^/Al_2_O_3_	40	Reflux for 4 h	49^b^
5	3 wt.% SO_4_^2−^/Al_2_O_3_	40	Stir at RT for 18 h	43
6	9 wt.% SO_4_^2−^/Al_2_O_3_	40	Reflux at for 4 h	< 5
7	MsOH (15 mmol)	40	Stir at RT for 18 h	27

Reaction conditions: 1.5 g of crude d-lyxonolactone (10 mmol) in 40 mL of acetone, 3 mmol of catalyst. Molar ratio of 2,3-acetonide **9a** to 3,5-acetonide **9b**: ^a^4:1. ^b^3:1.

## Conclusion

The novel strategy for synthesis of the iminosugars 1,5-dideoxy-1,5-imino-ribitol and 1,5-dideoxy-1,5-imino-dl-arabinitol synthesis was tested out in an environment friendly route starting from naturally occurring d-ribose and d-lyxose. Using ultra small gold clusters on Al_2_O_3_ (Au/Al_2_O_3_) could effectively oxidize d-ribose and d-lyxose to corresponding lactones under alkaline-free condition. SO_4_^2−^/Al_2_O_3_ showed good catalytic activities to replace homogenous Concentrated HCl and MsOH catalysts for acetalization of d-ribonolactone and d-lyxonolactone. The synthetic route resulted in good overall yields of 1,5-dideoxy-1,5-imino-d-ribitol of 54%, 1,5-dideoxy-1,5-imino-d-arabinitol of 48% and 1,5-dideoxy-1,5-imino-l-arabinitol of 46%. In addition, the heterogeneous catalysts and reagents applied in this synthetic strategy show environmental benefit and utilize the principles of green chemistry.

## Experimental section

See experiment detail in supplemental information.

## Supplementary Information

Supplementary Information.
