# Supplementary material for: Green synthesis of 1,5-dideoxy-1,5-imino-ribitol and 1,5-dideoxy-1,5-imino-dl-arabinitol from natural d-sugars over Au/Al2O3 and SO42−/Al2O3 catalysts
Source: Sci Rep. 2021 Aug 19;11:16928. doi: 10.1038/s41598-021-96231-9 (PMC8376872; doi:10.1038/s41598-021-96231-9)

Supplementary information

Green synthesis of 1,5-dideoxy-1,5-imino-ribitol and 1,5-dideoxy-1,5-imino-DL-arabinitol from natural D-sugars over Au/Al_2O_3 and SO_4^{2-}/Al_2O_3 catalysts

Hongjian Gao, Ao Fan*

Western Digital Corporation, 5601 Great Oaks Parkway,
San Jose, CA, USA 95119-1003
Email: fanao2013cn@gmail.com

Contents

Experimental Section..3

Preparation and characterization of catalysts..3

Identification of products..5

General procedure for oxidation of D-ribose **1** to D-ribonolactone **2** over Au/Al_2O_3 catalyst…....6

General procedure for one-pot transformation of D-ribonolactone **2** to mesylate **3**........................6

General procedure for epimerization of mesylate **3** to 1,5-dideoxy-1,5-imino-ribitol **4**.................7

General procedure for reduction of mesylate **3** to mesylate **5**..8

General procedure for hydrolysis of mesylate **5** to 1,5-dideoxy-1,5-imino-L-arabinitol **6**.............9

General procedure for oxidation of D-lyxose **7** to D-lyxonolactone **8** with Au/Al_2O_3 catalyst......9

General procedure for one-pot transformation of D-lyxonolactone **8** to mesylate **9**......................10

General procedure for epimerization of mesylate **9** to 1,5-dideoxy-1,5-imino-D-arabinitol **10**....11

1H NMR (300 MHz, DMSO-6) of crude D-ribonolactone and D-lyxonolactone **……**................12

1H NMR (300 MHz, $CDCl_3$) of mesylate **3**……………………………......................................13

13C NMR (300 MHz, acetone-d6) of mesylate **3**……………………….....................................13

1H NMR (300 MHz, D_2O) of 1,5-dideoxy-1,5-imino-ribitol **4……..**.......................................14

13C NMR (300 MHz, D_2O) of 1,5-dideoxy-1,5-imino-ribitol **4**..14

1H NMR (300 MHz, acetone-d6) of mesylate **5**...15

13C NMR (300 MHz, acetone-d6) of mesylate **5**...15

1H NMR (300 MHz, D_2O) of 1,5-dideoxy-1,5-imino-L-arabinitol **6**..16

13C NMR (300 MHz, D_2O) of 1,5-dideoxy-1,5-imino-L-arabinitol **6**..16

13C NMR (300 MHz, acetone-d6) of mesylate **9**...17

1H NMR (300 MHz, D_2O) of 1,5-dideoxy-1,5-imino-D-arabinitol **10**...18

13C NMR (300 MHz, D_2O) of 1,5-dideoxy-1,5-imino-D-arabinitol **10**.......................................18

Experimental Section

1.1 Chemical

All the chemicals were commercially available as reagent grade and used as received without further purification: γ-Al_2O_3 (20 nm) was obtained from Aladdin Industrial Corporation, ethanol (AR 99.7%, Adamas), ammonium hydroxide (AR, 25–28%, Adamas), D-ribose, D-lyxose (Carbosynth). Ultrapure water (18.2 MΩ cm) was produced using a Millipore water purification system and used for all solution preparations.

1.2 Preparation and characterization of catalysts

The Au/C with 3 wt.% gold loading and commercial Pd (10 wt.%)/C were purchased from Haruta Gold Incorporated and Degussa, respectively. 10 wt.% Pd-Bi/C was prepared according to the method we reported previously [1]. Au/Al_2O_3 catalyst was synthesized with solid grinding method [2]: γ-Al_2O_3 (3.0 g), [Me2Au(acac)] (acac=acetylacetonate) (50 mg), and acetone (13 ml) were ground by ball milling (350 rpm) at room temperature for 1 h. The resulting mixture was calcined in air at 300 ^oC for 4 h. Prior to use, the catalysts were reduced under H_2 flow for 2 h at 150 ^oC.

SO_4^{2-}/Al_2O_3 was synthesized by incipient wetness method [3]: γ-Al_2O_3 were impregnated with different concentration of aqueous H_2SO_4 solutions (15 mL solution/g γ-Al_2O_3 powder). The surface wt.% of SO_4^{2-}/Al_2O_3 was analyzed by XRF. For example, to prepare 3 wt.% SO_4^{2-}/Al_2O_3, 1 g of γ-Al_2O_3 powder was suspended in 1 M aqueous H_2SO_4 solution for 12 hour with continuous stirring at room temperature. Then the precipitate was dried at 60 ^oC for 24 h, followed by further drying at 100 ^oC for 24 h. Finally, the resulting solid was calcined in a stream of dry air at 500 ^oC for 24 h.

The recycle procedure for used SO_4^{2-}/Al_2O_3 catalyst is: after reaction, the mixture was cooled to room temperature and filtered. The resulting solid was calcined in a stream of dry air at 500 ^oC for 24 h.

The re-generation procedure for used SO_4^{2-}/Al_2O_3 catalyst is: after reaction, the mixture was cooled to room temperature and filtered. The resulting solid was calcined in a stream of dry air at 500 ^oC for 24 h. The recycled sample was then suspended in 0.5 M aqueous H_2SO_4 solution for 12 hour with continuous stirring at room temperature, followed by drying at 60 ^oC for 24 h and further drying at 100 ^oC for 24 h. Finally, the resulting solid was calcined in a stream of dry air at 500 ^oC for 24 h. Figure S1 shows NH_3-TPD curves of fresh, recycled and re-generated 3 wt.% SO_4^{2-}/Al_2O_3 catalysts.

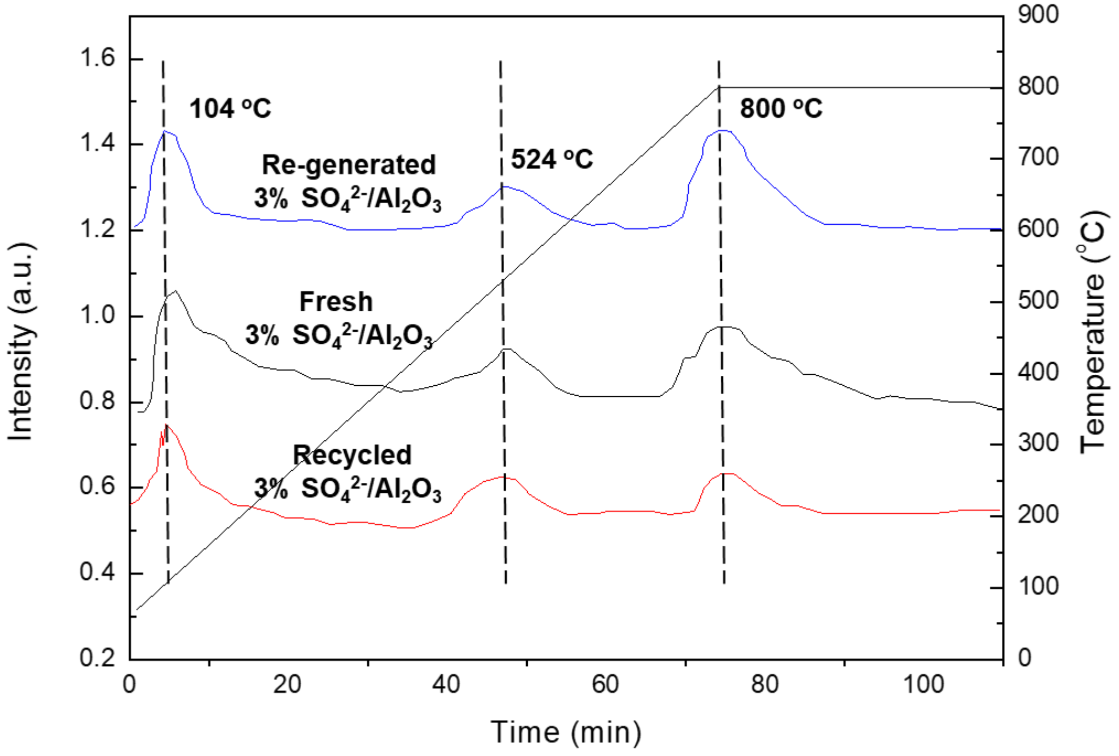

Figure S1. NH_3-TPD curves of fresh, recycled and re-generated 3 wt.% SO_4^{2-}/Al_2O_3 catalysts

The nitrogen adsorption/desorption isotherms of the Au/Al_2O_3 samples were measured with a Micromeritics Tristar 3000. Before the measurement, the sample was degassed at 100 °C for 6 h to remove physisorbed water. The surface area was determined using the Brunauer–Emmett–Teller (BET) method. The pore size distribution was calculated from the desorption branch of the isotherm using the Barrett–Joyner–Halenda (BJH) equation. The total pore volume of the sample was taken from the volume of nitrogen adsorbed at the P/P^o of 0.99. The crystal structure and phase of the samples were determined with a Siemens D5005 powder x-ray diffractometer equipped with a Cu anode and variable primary and secondary beam slits. The diffractograms were measured from 2 theta of 5° to 120°, using a step size of 0.02° and a dwell time of 1 s/step. X-ray photoelectron spectroscopy (XPS) was used to determine the surface elemental composition. The measurements were made with a VG Escalab MkII using monochromated Al Kα radiation (1486.6 eV, 15 kV) under a vacuum of 3×10^{-8} Pa. The binding energies are referenced to the carbon 1 s peak of CH at 284.6 eV. The peak areas were determined after peak fitting and normalized with the manufacturer's atomic sensitivity factors for the different elements. Au particle size on Al_2O_3 support was analyzed with scanning transmission electron microscopy (STEM, JEOL 2200FS),

The samples for this analysis were prepared by ultrasound dispersion in isopropanol and a drop of the solution was put on a carbon grid. The average size of Au particles and its distributions was estimated by counting about 300 Au particles. Inductively coupled plasma optical emission spectrometer (ICP-OES, Varian 720-ES instrument) was utilized to determine the Au content.

The XRF analysis was applied to identify the elements ratio of S/Al for the SO_4^{2-}/Al_2O_3 catalysts and was set at 30 kV and 0.120 mA with a live time of 110 sec. The acidity of the SO_4^{2-}/Al_2O_3 catalyst was examined with the ammonia temperature programmed desorption technique (NH_3-TPD). Firstly, the catalyst powder was placed in a quartz reactor and pretreated at 400 °C under helium (He) flow (30mL/min) for 0.5 h to remove any impurities. Then, the catalyst was cooled to 60 °C and equilibrated with NH_3. Thirdly, the catalyst was purged by He for 1 h to remove physically and gas-phase adsorbed NH_3. Once a stable baseline of NH_3 was acquired, the temperature was increased to 800 °C at the speed of 10 °C/min in He flow (30 mL/min) to allow the desorption of NH_3. The temperature was maintained at 800 °C for 40 min, and the amount of desorbed NH_3 was recorded by using the Balzers Prisma quadrupole mass spectrometer. The Brønsted to Lewis acid sites of SO_4^{2-}/Al_2O_3 catalyst was detected through pyridine-adsorption infrared spectroscopy. In general, two peaks at around 1450 and 1540 cm^{-1} are attributed to Lewis and Brønsted acid sites respectively, while the peak at 1490 cm^{-1} is assigned to both sites. This measurement was conducted with a Nicolet IS-10 spectrometer (100 scans, 4 cm^{-1} resolution, Thermofish, America) on thin wafers (10 mg/cm^2) prepared under 7×10^3 kPa pressure and pretreated *in-situ* in the IR cell. The IR spectrum was obtained at 25 °C after the pretreatment period and pyridine thermo-desorption in vacuum at increasing temperatures up to 150 °C.

References:

[1] A. Fan, S. Jaenicke, G.K. Chuah, *Org. Biomol. Chem.*, 2011, **9**, 7720-7726.

[2] T. Ishida, N. Kinoshita, H. Okatsu, T. Akita, T. Takei and M. Haruta, *Angew. Chem., Int. Ed.*, 2008, **47**, 9265–9268.

[3] M. Marczewski, A. Jakubiak, H. Marczewska, A. Frydrych, M. Gontarz and A. Snieguła, *Phys. Chem. Chem. Phys.*, 2004, **6**, 2513-2522.

1.3 Identification of products

Melting points were determined with a Buchi 535 melting point apparatus and were uncorrected. Proton and 13C NMR spectra were measured at 300 MHz with a Bruker Avance 300 NMR spectrometer using tetramethylsilane (TMS) as the internal standard. Chemical shifts were reported

in ppm downfield from TMS. Mass spectrometry (MS) and high resolution-mass spectrometry electron ionization (HR-MS EI） were taken with a Finnigan MAT95XL-T and Micromass VG7035 double focusing mass spectrometer of high resolution, respectively. Optical rotations were measured by a Perkin Elmer 341 polarimeter in a 1 dm cell. Analytical and preparative thin layer chromatography (TLC) were conducted on precoated TLC plates (silica gel 60 F254, Merck).

2.1 General procedure for oxidation of D-ribose 1 to D-ribonolactone 2 over Au/Al_2O_3 catalyst

A 20 mL pressure reactor was charged with 36 mg of 1 wt.% Au/Al_2O_3 catalyst, 0.15 g of D-ribose and 10 mL deionized water. The system was first vacuumed with a pump and then fed with oxygen (1 MPa). The reaction system was heated to 100 °C and kept for 2 h. After reaction, the reactor was rapidly immersed in a water bath to cool, and oxygen was released at the same time to stop the reaction. Afterward, the reaction mixtures were immediately syringed out, filtered and analyzed by high-performance liquid chromatography HPLC (Shimadzu SPD-10AV equipped with a UV-visible detector, 200 nm). A Jordi Gel DVB organic acid column (250 mm length x 10 mm diameter) was used with 0.05 M H_2SO_4 as the eluent (flow rate 1.5 ml min^{-1}). Figure S2 shows a representative HPLC spectrum of the reaction. In the recycle tests, the catalysts were recovered after reaction by centrifugation for 5 min (6000 rpm). The catalysts were washed with distilled water several times until the supernatant became neutral. The washed solids were dried at 80 °C for 2 h. Prior to reuse, the reused catalysts were reduced under H_2 flow for 2 h at 150 °C.

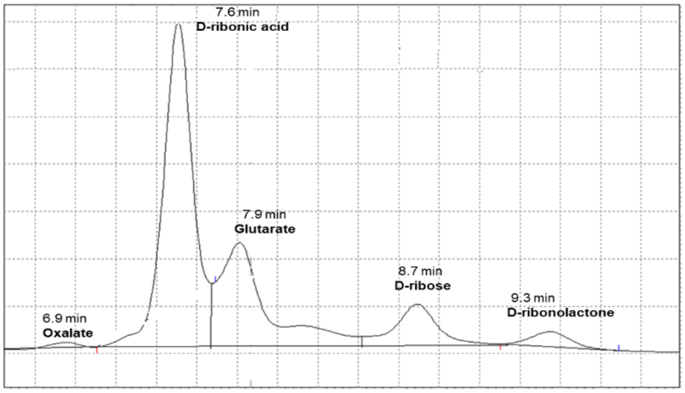

Figure S2 Representative HPLC spectrum of the reaction

2.2 General procedure for conversion of D-ribonolactone 2 to mesylate 3

The crude D-ribonolactone (1.5 g, obtained from oxidation of 1.5 g D-ribose) was dissolved in acetone (40 mL) and 3 wt.% SO_4^{2-}/Al_2O_3 (0.5 g) was added to the resulting solution. After stirred and refluxed for 2 h, the mixture was cooled to room temperature and filtered. The precipitate was

washed with hot acetone (10 mL). The filtrate together with the washing was rotary evaporated to give white solid. The white solid was dissolved in ethyl acetate (20 mL) and washed with deionized water (2 x 10 mL). The organic phase was rotary evaporated to dryness to afford 1.52 g of white crystals (2,3-*O*-isopropylidene-D-ribonolactone, 81 % overall yield from D-ribose, the characterization info of this pure product could be found in [4]). Then, the white crystals were dissolved in an ice-cooled pyridine (5 mL) and methanesulfonyl chloride (0.7 mL, 9 mmol) was added dropwise to the solution. The mixture was kept for 2 h at 0 °C. The reaction was quenched with water (5 mL) and CH_2C1_2 (15 mL) was added. The mixture was washed successively with 10 % aq HC1 (3 mL) until the extract became acidic and then with an additional portion of 10 % aq HC1 (3 mL) followed by aq $NaHCO_3$ (3 mL). The organic phase was dried with $MgSO_4$, then treated with activated carbon, filtered. The filtrate was concentrated in vacuo to give **3** as colorless crystals (1.93 g, 73 % overall yield from D-ribose).

Mp 65-66 °C; $[\alpha]_D^{20}$ = - 49.4 °C (c = 1.0, $CHCl_3$); ^1H NMR (300 mHz, $CDCl_3$): δ 4.82 (1H, d, $J_{2,3}$ = 6.1 Hz, H-2), 4.79 (1H, dd, $J_{3,4}$ = 1.2 Hz, H-3),4.77 (1H, t, $J_{4,5}$ = 2.4, $J_{4,5'}$ = 2.4 Hz, H-4), 4.47 (1H, dd, $J_{5,5'}$ =11.2 Hz, H-5'), 4.40 (1H, dd, H-5), 3.03 (3 H, Ms), 1.47 (3 H, s, CH_3), 1.40 (3 H, s, CH_3); ^{13}C NMR (300 mHz, acetone-d6): 172.9 (C-1), 113.6 (acetal C), 79.6 (C-4), 77.5 (C-3), 75.1 (C-2), 68.4 (C-5), 37.6 (mesyl), 26.6, 25.4 (CH_3).

Compare [5]: Mp 66-67 °C; $[\alpha]_D^{20}$ = - 49.9 °C (c = 1.0, $CHCl_3$); ^1H NMR (500 mHz, $CDCl_3$): δ 4.82 (1H, d, $J_{2,3}$ = 6.0 Hz, H-2), 4.79 (1H, dd, $J_{3,4}$ = 1.0 Hz, H-3),4.77 (1H, t, $J_{4,5}$ = 2.5, $J_{4,5'}$ = 2.5 Hz, H-4), 4.47 (1H, dd, $J_{5,5'}$ =11.5 Hz, H-5'), 4.44 (1 H, dd, H-5), 3.05 (3 H, Ms), 1.49 (3 H, s, CH_3), 1.40 (3 H, s, CH_3); ^{13}C NMR (500 mHz, acetone-d6): 173.1 (C-1), 113.8 (acetal C), 79.1 (C-4), 77.2 (C-3), 74.8 (C-2), 68.1 (C-5), 37.4 (mesyl), 26.5, 25.3 (CH_3).

[4] http://www.rsc.org/suppdata/ob/c1/c1ob06116j/c1ob06116j.pdf

[5] H. Kold, I. Lundt, C. Pedersen, *Acta. Chem. Scand.*, 1994, **48**, 675-678.

2.3 General procedure for conversion of mesylate 3 to 1,5-dideoxy-1,5-imino-ribitol 4

Mesylate **3** (1.0 g, 3.80 mmol) was dissolved in aq NH_3 (5 mL, 25%) and allowed to stand for 18 h at room temperature in a sealed flask. Concentration and co-concentration twice with EtOAc gave a residue which was extracted with boiling EtOAc (2 x 15 mL). The combined organic phases were treated with activated carbon, dried with Na_2SO_4, filtered. The filtrate was concentrated in vacuo to 0.6 g colorless crystals. The crystals were then dissolved in methanol (15 mL) and the resulting solution was cooled to -20 °C and $NaBH_4$ (0.26 g, 6.5 mmol) was slowly added over 1 h. After

stirring for 4 h, the solution was adjusted to pH 5-6 with 1 M HCl solution. Concentration in vacuo afforded a white solid. The white solid was suspended in CH_2Cl_2 (20 mL) and the suspension was heated to boiling. The hot suspension was filtered and the solids were rinsed with hot CH_2Cl_2 (10 mL). The filtrate was concentrated in vacuo to give a 0.48 g syrup. The syrup was dissolved in water (20 ml) and Amberlite IR-120H (3.0 g) was added to the solution. The mixture was kept at room temperature and stirred overnight. After filtering out the ion exchange resin, the resin was eluted with 5-15% aqueous NH_3. The resulting eluent was concentrated under reduced pressure.1,5-dideoxy-1,5-imino-D-ribitol **4** was isolated as the HCl salt (0.49 g, 2.88mmol, 76 % overall yield from Mesylate **3**).

Mp 184-186°C; $[\alpha]_D^{20}$ =0.0 ° (c = 1.0, H_2O); ^{1}H NMR (300 mHz, D_2O): δ 4.03 (2 H, ddd, $J_{1,2}$ =$J_{4,5}$ = 4.4 Hz, $J_{1,2}$ = $J_{4,5'}$ = 7.0 Hz, $J_{2,3}$ = $J_{3,4}$ = 2.6 Hz, H-2, H-4), 3.96 (1 H, t, H-3), 3.22 (2 H, dd, $J_{1,1'}$ = $J_{5,5'}$ = 12.8 Hz, H-l, H-5), 3.15 (2 H, dd, H-I', H-5'); ^{13}C NMR (300 mHz, D_2O): δ 69.5 (C-3), 66.3 (C-l, C-5), 45.6 (C-2, C-4); HRMS (ESI) m/z calcd. for [C5H11O3N+H]+: 134.0817, found: 134.0813.

Compare [6]: Mp 185-186.5 °C; $[\alpha]_D^{20}$ = 0.0 ° (c =1.0, H_2O); ^{1}H NMR (500 mHz, D_2O): δ 4.08 (2 H, ddd, $J_{1,2}$ =$J_{4,5}$ = 4.5 Hz, $J_{1,2}$ = $J_{4,5'}$ = 7.2 Hz, $J_{2,3}$ = $J_{3,4}$ = 2.9 Hz, H-2, H-4), 3.96 (1H, t, H-3), 3.26 (2H, dd, $J_{1,1'}$ = $J_{5,5'}$ = 13.0 Hz, H-l, H-5), 3.22 (2 H, dd, H-1', H-5'); ^{13}C NMR (500 mHz, D_2O): δ 69.1 (C-3), 66.3 (C-l, C-5), 45.0 (C-2, C-4).

[6] M. Godskesen, I. Lundt, R. Madsen, B. Winchester, *Bioorg. Med. Chem.*, 1996, **11**, 1857-1865.

2.4 General procedure for conversion of mesylate 3 to mesylate 5

To mesylate **3** (2.12g, 8 mmol) was added a solution of KOH (1.3 g, 23.2 mmol, 2.9 equiv) in water (10 mL), keeping the temperature at 25 °C. After stirring for 6 h, the pH was adjusted to 2.5-3.0 by adding 1 M HCl. The acidic solution was concentrated in vacuo to afford a solid mass. The solid mass was triturated with acetone (15 mL) and heated to reflux for 15 min. The acetone was decanted and the procedure was repeated. The combined acetone was dried over Na_2SO_4, and filtered. The clear filtrate was concentrated in vacuo below 35 °C to afford white crystals. Then, the white crystals were dissolved in an ice-cooled pyridine (5 mL) and methanesulfonyl chloride (0.7 mL, 9 mmol) was added dropwise to the solution. The mixture was kept for 2 h at 0 °C. The reaction was quenched with water (5 mL) and CH_2C1_2 (15 mL) was added. The mixture was washed successively with 10% aq HC1 (3 mL) until the extract became acidic and then with an additional portion of 10% aq HC1 (3 mL) followed by aq $NaHCO_3$ (3 mL). The organic phase was dried with

$MgSO_4$, then treated with activated carbon, filtered and concentrated to give **5** as colorless crystals (1.70 g, 80 %).

Mp 132-133 °C; $[\alpha]_D^{20}$ = -75.6 ° (c =1.0, CH_3OH); ^{1}H NMR (300 mHz, acetone-d6): δ 5.19 (1H, d, $J_{2,3}$ = 6.5 Hz, H-2), 4.91 (1 H, dd, $J_{3,4}$ = 4.0 Hz, H-3), 4.76 (1 H, ddd, $J_{4,5}$ = 8.0 Hz, H-4), 4.50 (1 H, dd, H-5), 4.32 (1 H, d, H-5'), 3.10 (3 H, Ms), 1.43 (3 H, s, CH_3) and 1.38 (3 H, s, CH_3); ^{13}C NMR (300 mHz, acetone-d6): δ 173.6 (C-1), 114.3 (acetal C), 78.2 (C-4), 77.8 (C-2), 77.8 (C-3), 65.1 (C-5), 38.3 (Ms), 27.3 and 26.1 (2 x CH_3).

Compare [6]: Mp 133-133.5°C; $[\alpha]_D^{20}$ = -75.9 ° (c = 1.0, CH_3OH); ^{1}H NMR (500 mHz, acetone-d6): δ 5.11 (1 H, d, $J_{2,3}$ = 6.3 Hz, H-2), 5.05 (1 H, dd, $J_{3,4}$ = 3.8 Hz, H-3), 4.95 (1 H, ddd, $J_{4,5}$ = 8.2 Hz, H-4), 4.65 (1 H, dd, H-5), 4.45 (1 H, d, H-5'), 3.19 (3 H, Ms), 1.42 (3 H, s, CH_3) and 1.36 (3 H, s, CH_3); ^{13}C NMR (500 mHz, acetone-d6): δ 173.9 (C-1), 114.6 (acetal C), 77.4 (C-4), 77.0 (C-2), 77.0 (C-3), 68.9 (C-5), 37.4 (Ms), 26.9 and 25.9 (2 x CH_3).

[6] M. Godskesen, I. Lundt, R. Madsen, B. Winchester, *Bioorg. Med. Chem.*, 1996, **11**, 1857-1865.

2.5 General procedure for the conversion of mesylate 5 to 1,5-dideoxy-1,5-imino-L-arabinitol 6

Followed the same experiment and work-up procedure as described in **2.3** to obtain 1,5-dideoxy-1,5-imino-L-arabinitol **6** as the HCl salt (78 % overall yield from Mesylate 5).

Mp 194.5-195 °C; $[\alpha]_D^{20}$ = 22.4° (c = 0.8, CH_3OH); ^{1}H NMR (300 mHz, D_2O): δ 4.18 (1 H, m, H-4), 4.02 (1 H, dt, $J_{1,2}$ = 4.2 Hz, $J_{1',2}$ = 8.6 Hz, $J_{2,3}$ = 8.0 Hz, H-2), 3.73 (1 H, dd, H-3), 3.43 (1 H, ddd, $J_{1,1'}$ =12.6 Hz, $J_{1,5}$ = 1.0 Hz, H-1), 3.22 (1 H, ddd, H-5), 3.15 (1 H, dd, H-5') and 2.86 (1 H, dd, H-1); ^{13}C NMR (300 mHz, D_2O): δ 70.4 (C-3), 65.5 (C-2), 65.3 (C-4), 45.6 (C-5) and 45.2 (C-1); HRMS(ESI) m/z calcd. for [C5H11O3N+H]+:134.0817, found: 134.0812.

Compare [6]: Mp 195.5-196 °C; $[\alpha]_D^{20}$ = 22.7 ° (c = 0.8, CH_3OH); ^{1}H NMR (500 mHz, D_2O): δ 4.19 (1 H, m, $J_{3,4}$ = 3.0 Hz, $J_{4,5}$ = 6.0 Hz, $J_{4,5'}$ = 2.8 Hz, H-4), 4.05 (1 H, dt, $J_{1,2}$ = 4.0 Hz, $J_{1',2}$ = 8.5 Hz, $J_{2,3}$ = 8.0 Hz, H-2), 3.73 (1 H, dd, H-3), 3.37 (1 H, ddd, $J_{1,1'}$ =12.8 Hz, $J_{1,5}$ = 1.0 Hz, H-1), 3.25 (1 H, ddd, $J_{5,5'}$ = 13.0 Hz, H-5), 3.17 (1 H, dd, H-5') and 2.92 (1 H, dd, H-1); ^{13}C NMR (500 mHz, D_2O): δ 70.4 (C-3), 64.6 (C-2), 64.3 (C-4), 45.6 (C-5) and 45.1(C-1).

[6] M. Godskesen, I. Lundt, R. Madsen, B. Winchester, *Bioorg. Med. Chem.*, 1996, **11**, 1857-1865.

3.1 General procedure for oxidation of D-lyxose 7 to D-lyxonolactone 8 with Au/ Al_2O_3 catalyst

The above procedure for the oxidation of D-ribose to D-ribonolactone was adopted for the oxidation of D-lyxose (Carbosynth). Using HPLC, the retention time for D-lyxose is 8.4 min and that for D-lyxonic acid is 7.6 min and D-lyxonolactone is 9.5 min. After 2 h, all D-lyxose was consumed giving a yield for D-lyxonolactone of > 95 %.

3.2 General procedure for conversion of D-lyxonolactone 8 to mesylate 9

The crude D-lyxonolactone (1.50 g, obtained from the oxidation of 1.5 g D-lyxose) was suspended in acetone (40 mL) and 3% SO_4^{2-}/Al_2O_3 (0.5 g) was added to the resulting solution. After stirring for 18 h at room temperature, the mixture was filtered and the solid was rinsed with hot acetone (10 mL). The filtrate together with the washing was rotary evaporated to give a white solid. The white solid was dissolved in ethyl acetate (20 mL) and then washed twice with deionized water (10 mL). The organic phase was rotary evaporated to dryness to afford 0.76 g (2,3-O-isopropylidene-D-lyxonolactone, 41 % overall yield from D-lyxose) of white crystalline solid. The aqueous phase was rotary evaporated to dryness followed by adding 3% SO_4^{2-}/Al_2O_3 (0.5 g) and acetone (40 mL) and stirring at room temperature for 18 h. After subjecting the unreacted D-lyxonolactone in aqueous phase to another 2 cycles of reaction, a total of 1.41 g of white crystalline solid was obtained (76 % overall yield from D-lyxose, the characterization info of this pure product could be found in [4])). Then, the white crystals were dissolved in an ice-cooled pyridine (5 mL) and methanesulfonyl chloride (0.7 mL, 9 mmol) was added dropwise to the solution. The mixture was kept for 2 h at 0 °C. The reaction was quenched with water (5 mL) and CH_2C1_2 (15 mL) was added. The mixture was washed successively with 10% aq HC1 (3 mL) until the extract became acidic and then with an additional portion of 10% aq HC1 (3 mL) followed by aq $NaHCO_3$ (3 mL). The organic phase was dried ($MgSO_4$), treated with activated carbon, filtered. The filtrate was concentrated in vacuo to give **9** as colorless crystals (1.77 g, 67 % overall yield from D-lyxose).

Mp 126.5-127.5°C; $[\alpha]_D^{20}$ = -76.8 ° (c = 1.0, MeOH); ^{13}C NMR (300 mHz, acetone-d6): δ 172.9 (C-1), 113.6 (acetal C), 76.1, 75.3, 75.1 (C-2, 3, 4), 68.4 (C-5), 37.6 (Mesyl), 25.6 and 24.3 (2 x CH_3).

Compare [7]: Mp 127.5-128.5°C; $[\alpha]_D^{20}$ = -76.6 ° (c = 1.0, MeOH); ^{13}C NMR (300 mHz, acetone-d6): δ 173.9 (C-1), 114.6 (acetal C), 77.4 (C-4), 77.0 (C-2), 77.0 (C-3), 68.9 (C-5), 37.4 (Mesyl), 26.9 and 25.9 (2 x CH_3).

[7] M. Godskesen,I. Lundt, I. Søtofte, *Tetrahedron: Asymmetry*, 2000, **11**, 567–579.

3.3 General procedure for the conversion of mesylate 9 to 1,5-Dideoxy-1,5-imino-D-arabinitol 10

Followed the same experiment and work-up procedure as described in **2.3** to obtain 1,5-dideoxy-1,5-imino-D-arabinitol **10** as the HCl salt (72 % overall yield from Mesylate **9**).

Mp 190-191°C; $[\alpha]_D^{20}$ = -17.8 ° (c = 0.7, MeOH); ^{1}H NMR (300 mHz, D_2O): δ 3.93 (1H, ddd, H-4), 3.66 (1 H, ddd, H-2), 3.31 (1H dd, H-3), 3.01 (1H, dd, H-l'), 2.94 (1 H, dd, H-5'), 2.75 (1 H, dd, H-5), 2.60 (1H, dd, H-l); ^{13}C NMR (500 mHz, D_2O): δ 71.8 (C-3), 66.6, 66.1 (C-2, C-4), 47.4, 47.0 (C-1, C-5); HRMS (ESI) m/z calcd. for [C5H11O3N+H]+:134.0817, found: 134.0812.

Compare [8]: Mp 191-192°C; $[\alpha]_D^{20}$ = -18 ° (c = 0.7, MeOH); ^{1}H NMR (500 mHz, D_2O): δ 3.91 (1 H, ddd, H-4), 3.75 (1 H, ddd, H-2), 3.44 (1 H, dd, H-3), 3.09 (1 H, dd, H-l'), 2.94 (1 H, dd, H-5'), 2.75 (1 H, dd, H-5), 2.60 (1 H, dd, H-l); ^{13}C NMR (500 mHz, D_2O): δ 71.6 (C-3), 65.9, 65.5 (C-2, C-4), 47.0, 46.4 (C-1, C-5).

[8] G. Legler, A.E.Stütz, H. Immich, *Carbohydr. Res.*, 1995, **272**, 17-30.

Figure S3. 1H NMR (300 MHz, DMSO-d6) of crude D-ribonolactone

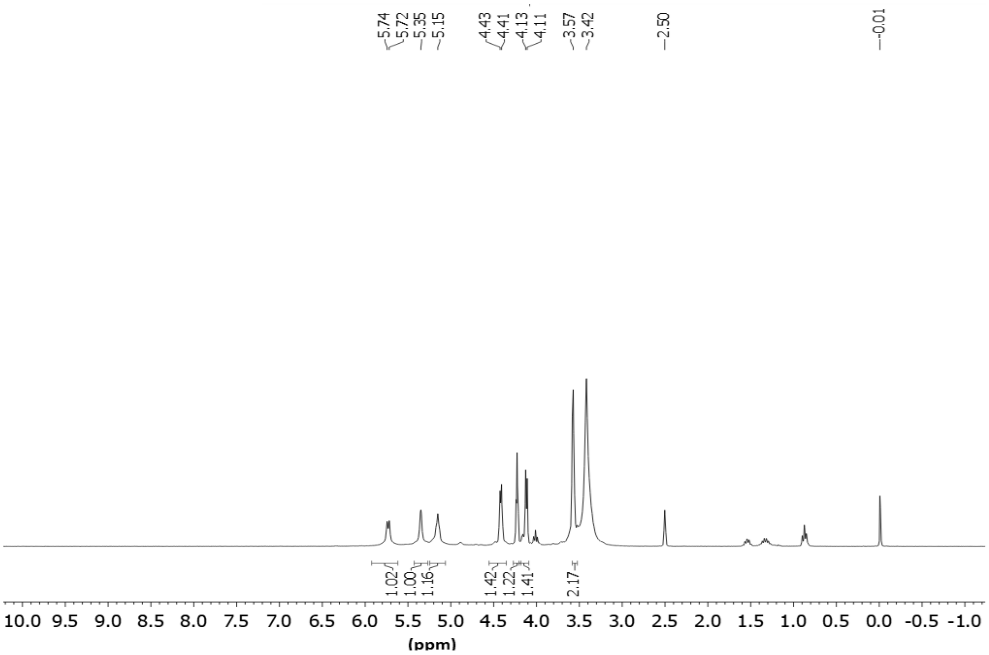

Figure S4. 1H NMR (300 MHz, DMSO-d6) of crude D-ribonolactone

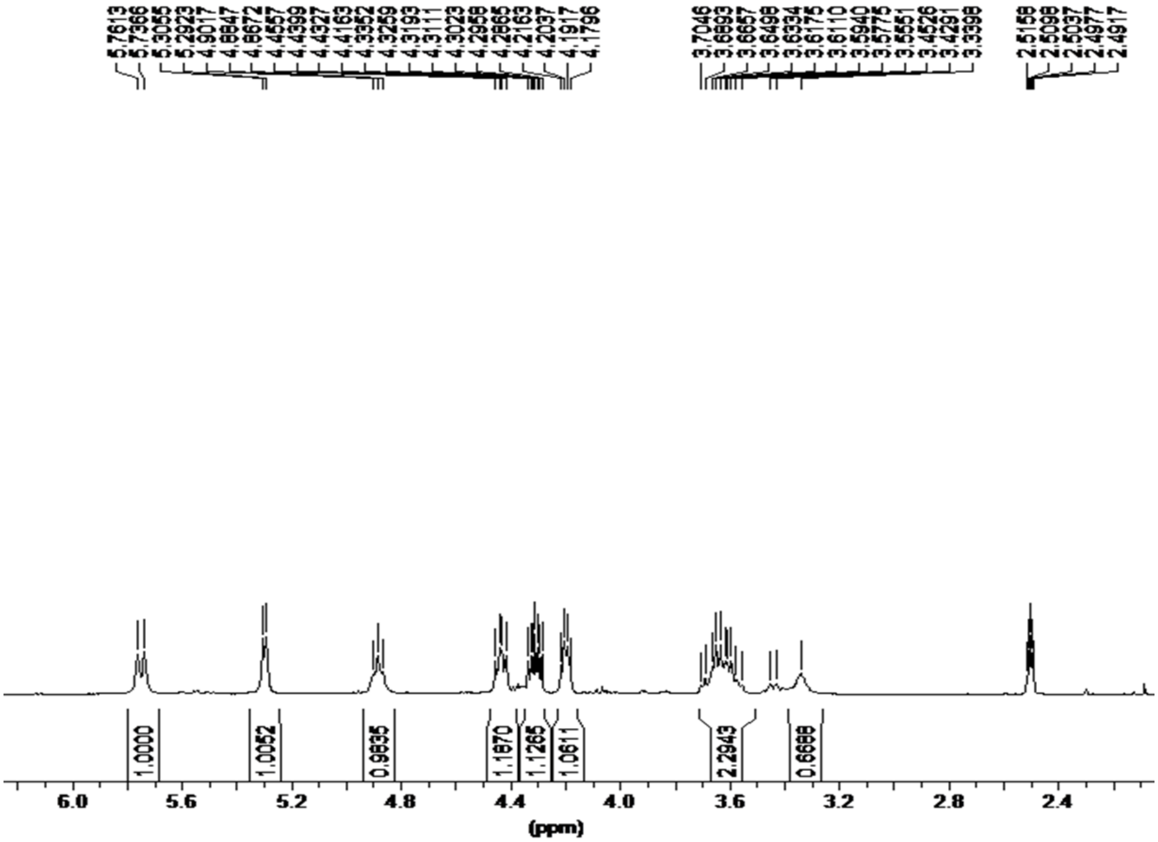

Figure S5. 1H NMR (300 MHz, $CDCl_3$) of mesylate **3**

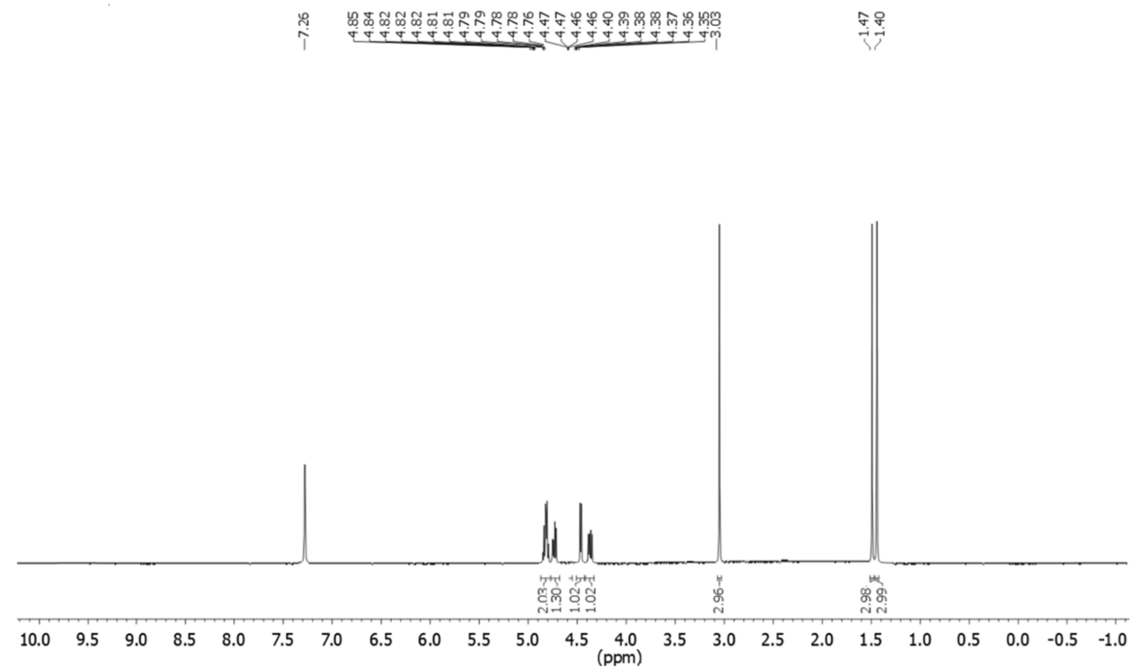

Figure S6.13C NMR (300 MHz, acetone-d6) of mesylate **3**

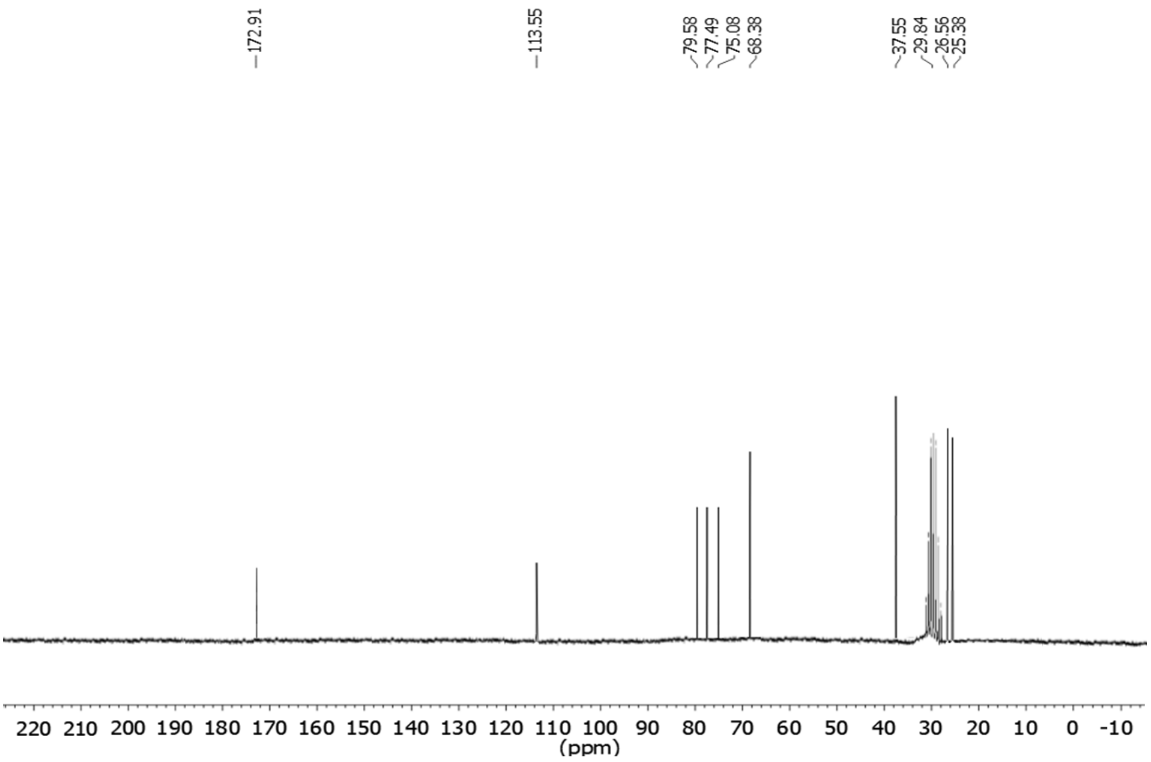

Figure S7. 1H NMR (300 MHz, D_2O) of 1,5-dideoxy-1,5-imino-ribitol **4**

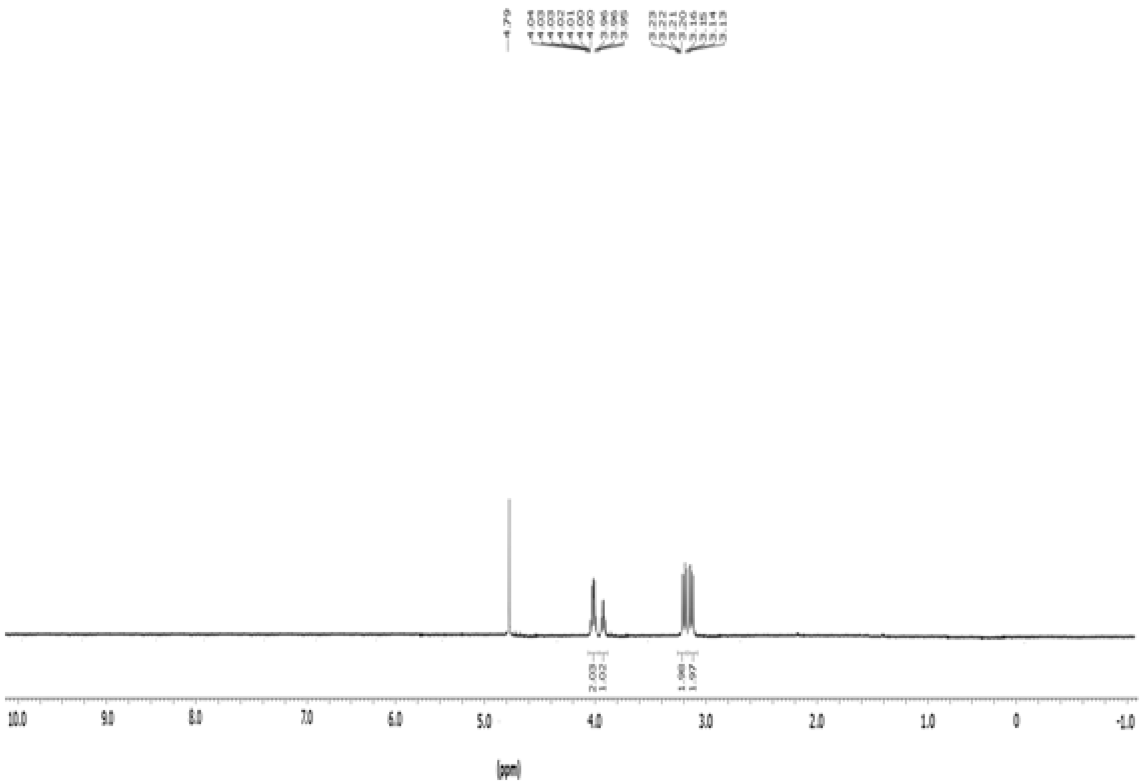

Figure S8. 13C NMR (300 MHz, D_2O) of 1,5-dideoxy-1,5-imino-ribitol **4**

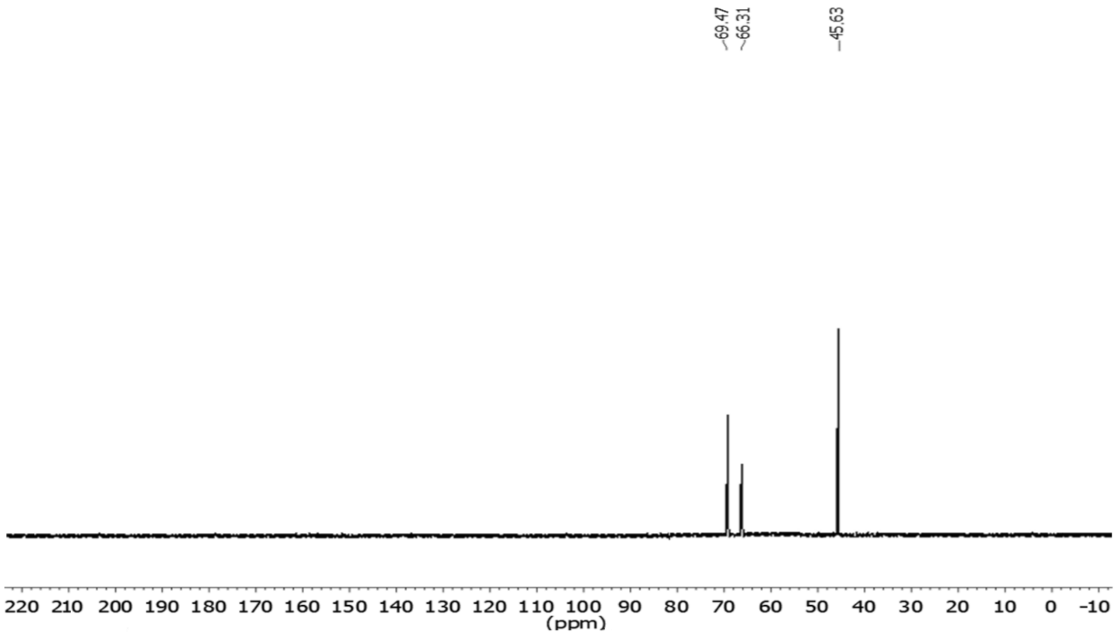

Figure S9. 1H NMR (300 MHz, acetone-d6) of mesylate **5**

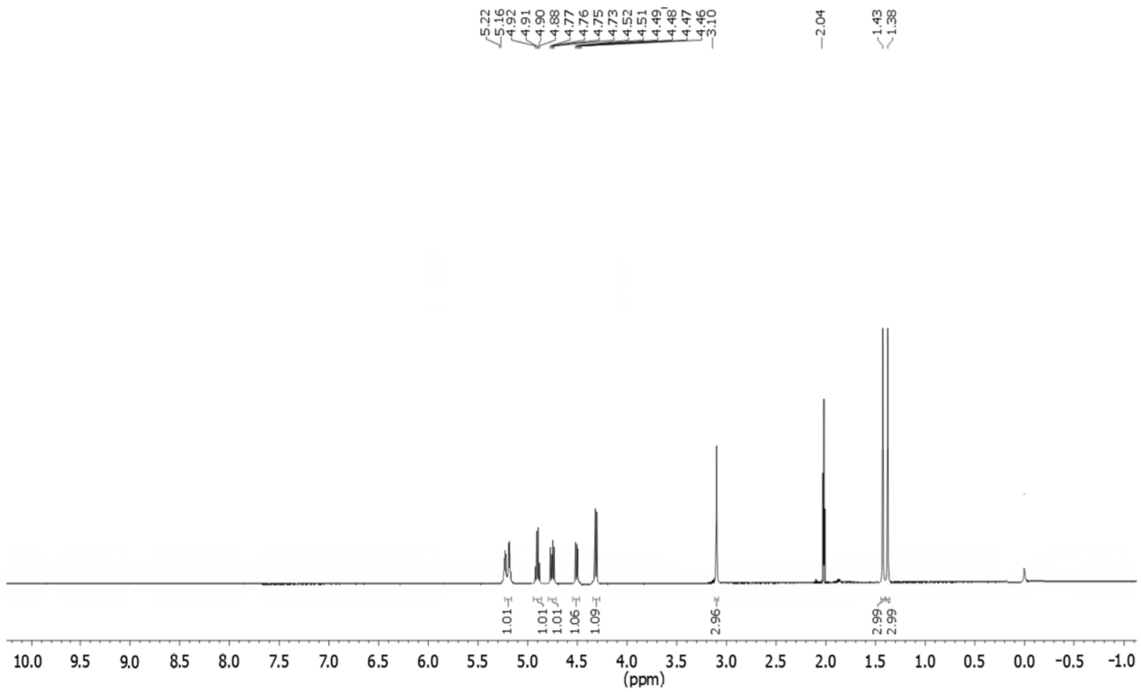

Figure S10. 13C NMR (300 MHz, acetone-d6) of mesylate **5**

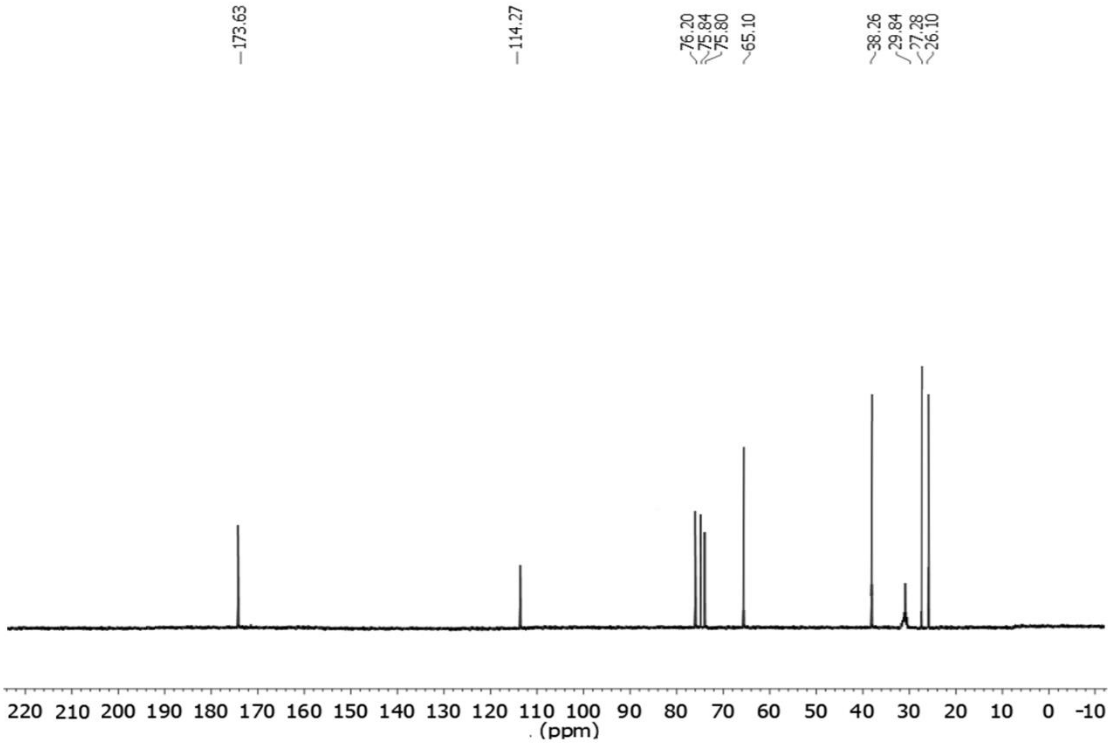

Figure S11. 1H NMR (300 MHz, D_2O) of 1,5-dideoxy-1,5-imino-L-arabinitol **6**

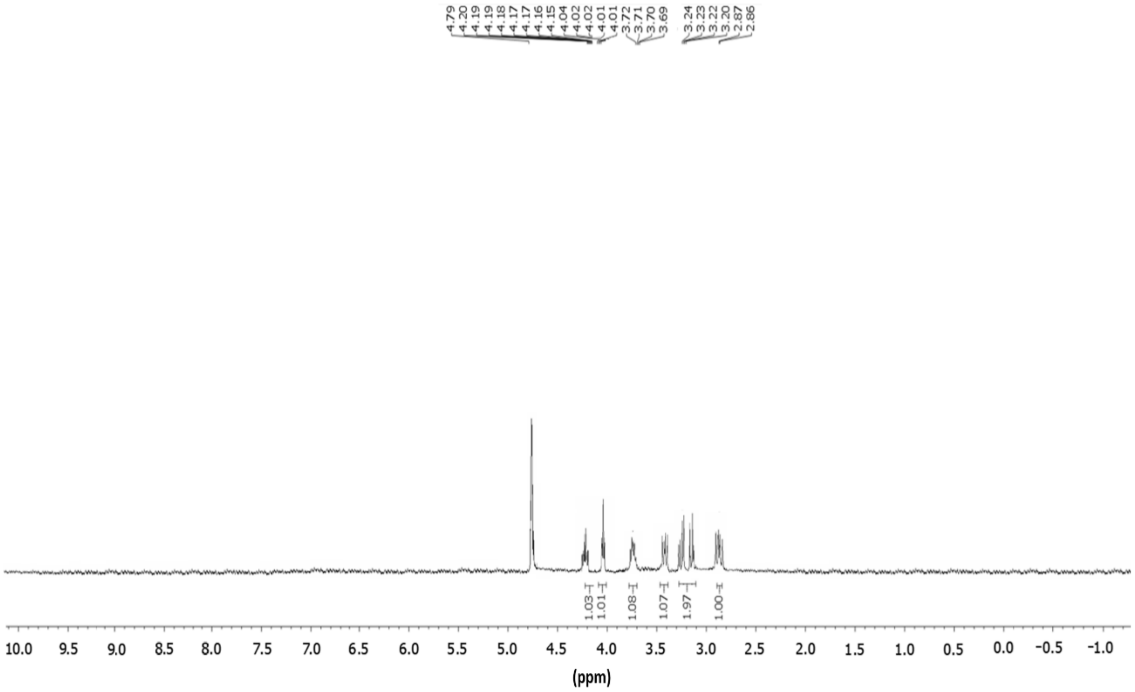

Figure S12. 13C NMR (300 MHz, D_2O) of 1,5-dideoxy-1,5-imino-L-arabinitol **6**

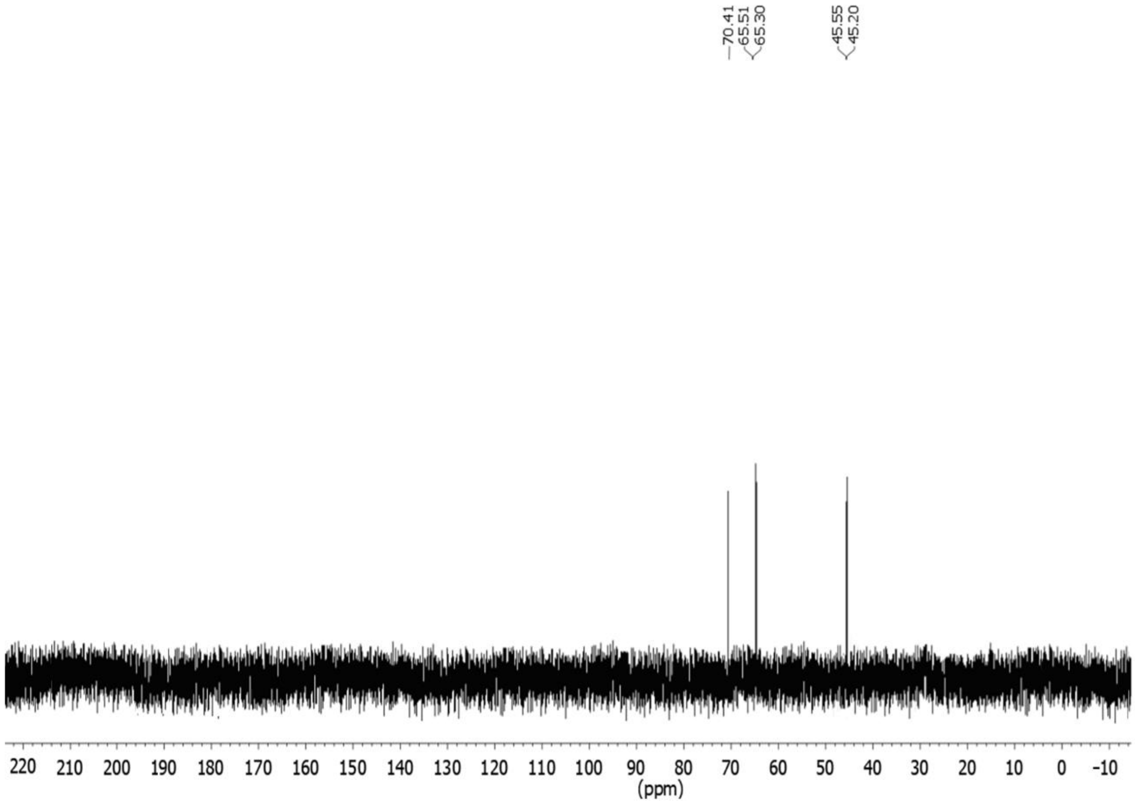

Figure S13. 13C NMR (300 MHz, acetone-d6) of mesylate **9**

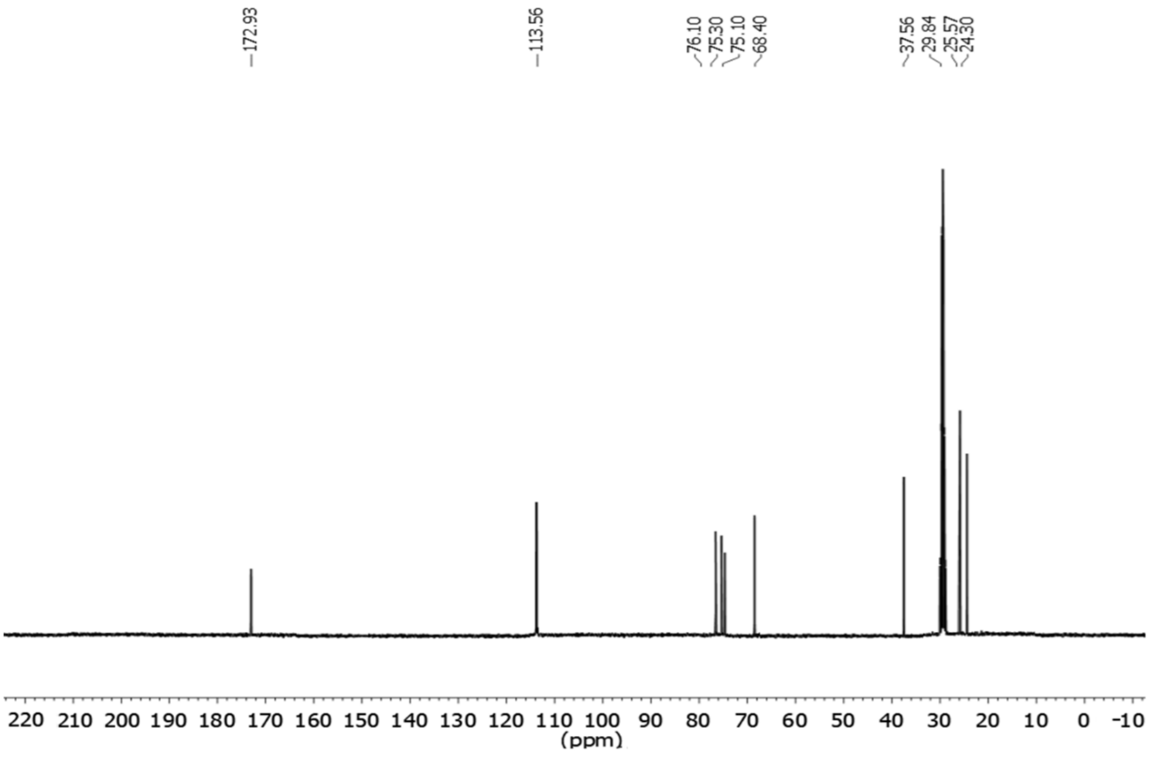

Figure S14. 1H NMR (300 MHz, D_2O) of 1,5-Dideoxy-1,5-imino-D-arabinitol **10**

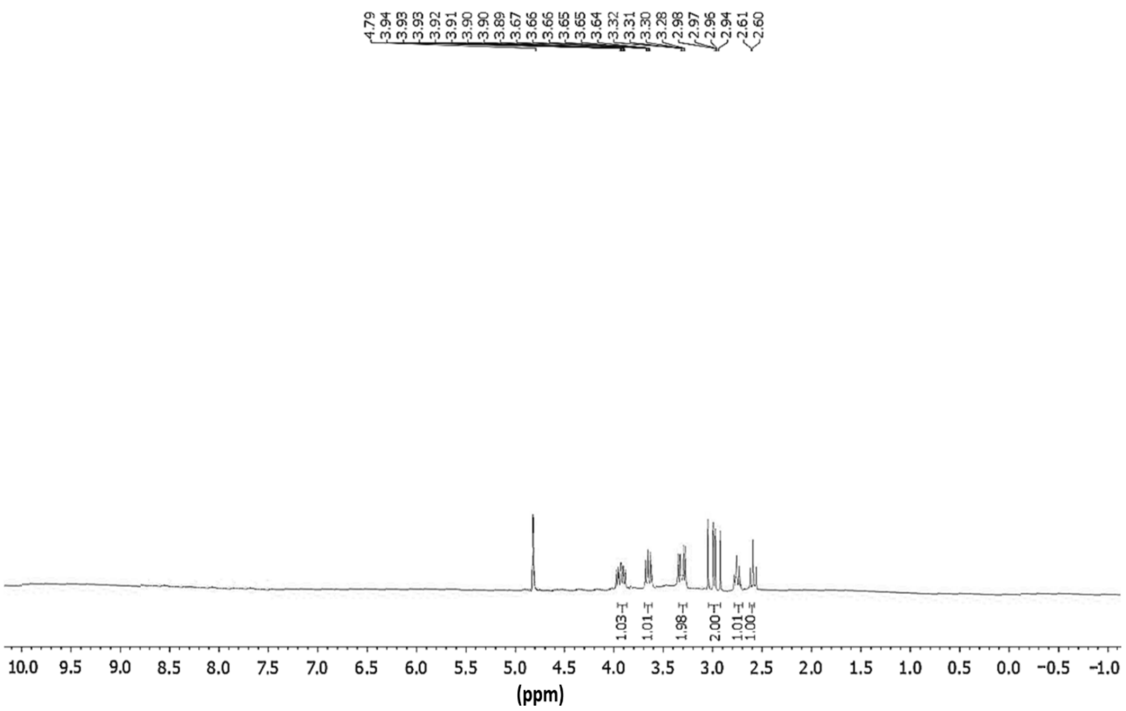

Figure S15. 13C NMR (300 MHz, D_2O) of 1,5-Dideoxy-1,5-imino-D-arabinitol **10**

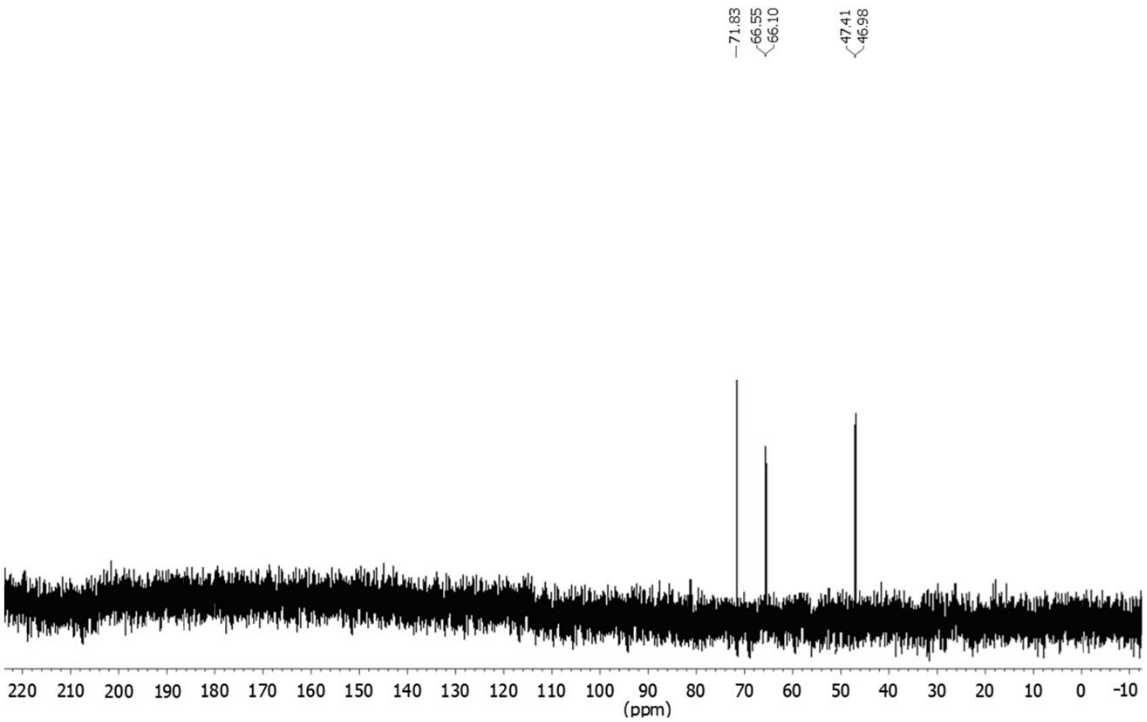

Supplement: Supplementary file 1 — Supplementary Information. [file 41598_2021_96231_MOESM1_ESM.pdf]
